# The human AP-endonuclease 1 (APE1) is a DNA G-quadruplex structure binding protein and regulates *KRAS* expression in pancreatic ductal adenocarcinoma cells

**DOI:** 10.1093/nar/gkac172

**Published:** 2022-03-14

**Authors:** Suravi Pramanik, Yingling Chen, Heyu Song, Irine Khutsishvili, Luis A Marky, Sutapa Ray, Amarnath Natarajan, Pankaj K Singh, Kishor K Bhakat

**Affiliations:** Department of Genetics, Cell Biology and Anatomy, University of Nebraska Medical Center, Omaha, NE 68198, USA; Department of Genetics, Cell Biology and Anatomy, University of Nebraska Medical Center, Omaha, NE 68198, USA; Department of Genetics, Cell Biology and Anatomy, University of Nebraska Medical Center, Omaha, NE 68198, USA; Department of Pharmaceutical Sciences, University of Nebraska Medical Center, Omaha, NE 68198, USA; Department of Pharmaceutical Sciences, University of Nebraska Medical Center, Omaha, NE 68198, USA; Hematology/Oncology Division, Department of Pediatrics, University of Nebraska Medical Center, Omaha, NE 68198, USA; Eppley Institute for Research in Cancer and Allied Health, University of Nebraska Medical Center, Omaha, NE 68198, USA; Fred & Pamela Buffett Cancer Center, University of Nebraska Medical Center, Omaha, NE 68198, USA; Eppley Institute for Research in Cancer and Allied Health, University of Nebraska Medical Center, Omaha, NE 68198, USA; Fred & Pamela Buffett Cancer Center, University of Nebraska Medical Center, Omaha, NE 68198, USA; Department of Genetics, Cell Biology and Anatomy, University of Nebraska Medical Center, Omaha, NE 68198, USA; Fred & Pamela Buffett Cancer Center, University of Nebraska Medical Center, Omaha, NE 68198, USA

## Abstract

Pancreatic ductal adenocarcinoma (PDAC), one of the most aggressive types of cancer, is characterized by aberrant activity of oncogenic *KRAS*. A nuclease-hypersensitive GC-rich region in *KRAS* promoter can fold into a four-stranded DNA secondary structure called G-quadruplex (G4), known to regulate *KRAS* expression. However, the factors that regulate stable G4 formation in the genome and *KRAS* expression in PDAC are largely unknown. Here, we show that APE1 (apurinic/apyrimidinic endonuclease 1), a multifunctional DNA repair enzyme, is a G4-binding protein, and loss of APE1 abrogates the formation of stable G4 structures in cells. Recombinant APE1 binds to *KRAS* promoter G4 structure with high affinity and promotes G4 folding *in vitro*. Knockdown of APE1 reduces MAZ transcription factor loading onto the *KRAS* promoter, thus reducing *KRAS* expression in PDAC cells. Moreover, downregulation of APE1 sensitizes PDAC cells to chemotherapeutic drugs *in vitro* and *in vivo*. We also demonstrate that PDAC patients’ tissue samples have elevated levels of both APE1 and G4 DNA. Our findings unravel a critical role of APE1 in regulating stable G4 formation and *KRAS* expression in PDAC and highlight G4 structures as genomic features with potential application as a novel prognostic marker and therapeutic target in PDAC.

## INTRODUCTION

Pancreatic ductal adenocarcinoma (PDAC) is the third leading cause of cancer-related deaths in the United States, with a 5-year survival rate of ∼9% (1). Being one of the most aggressive and lethal solid malignancies, PDAC is highly resistant to chemotherapy and radiation and remains a major clinical challenge (2,3). The proto-oncogene, *KRAS*, is the most frequently mutated oncogene in PDAC (∼95% of cases) (4,5). Activating point mutations at glycine-12, glycine-13 or glutamine-61 impair the intrinsic GTPase activity of KRAS protein and lead to its constitutive activation and persistent stimulation of the downstream signaling pathways that drive oncogenesis (6–10). There is growing evidence that the maintenance of the malignant phenotype depends on the constitutive expression of *KRAS*, a phenomenon termed ‘oncogene addiction’ (7,11–14). Consequently, suppression of mutated *KRAS* impacts the viability of pancreatic cancer cells. Thus, the absolute importance of mutant *KRAS* expression in PDAC has driven multiple investigations to target it both at the transcriptional and translational levels (5,15,16).

Several studies have demonstrated that the promoter region of many cancer-driving oncogenes, including *KRAS* and *c-MYC*, have G-rich DNA sequences that form a noncanonical, four-stranded nucleic acid structure called G-quadruplex (G4) (17,18). The G4 structures are highly abundant in the gene regulatory elements such as gene promoters, 5′ and 3′ untranslated regions, replication initiation sites and telomeres (19,20). They have been implicated in modulation of gene expression including that of oncogenes, alteration in chromatin states, and in the maintenance of telomere and genome stability (21). Recent genome-wide analysis revealed that G4 DNA structures are hubs for transcription factor (TF) binding (22). Because of their important regulatory functions pertaining to oncogene expression, G4 structure has emerged as a potential therapeutic target in cancer (23–26).

The core promoter region of the human *KRAS*, encompassed within the region from +50 to −510 bp with respect to the transcription start site (TSS), is highly G/C rich (75%) and can form three G4 structures (27–29). The proximal G4 that lies between −148 and −116 bp upstream of TSS overlaps with a nuclease-hypersensitive element and is recognized by several nuclear proteins. It was demonstrated that binding of TFs such as MAZ, hnRNP A1 and PARP-1 in this region activates *KRAS* expression (17,30–33). Furthermore, oxidative stress that induced guanine (G) oxidation in the regulatory G4 motif in *KRAS* was shown to upregulate *KRAS* expression (34). Despite these well-established roles of G4 in *KRAS* transcriptional regulation, little is known about the molecular machinery that regulates the formation and stability of these G4 structures in cells.

Recently, we demonstrated a genome-wide correlation between the occupancy of apurinic/apyrimidinic endonuclease 1 (APE1) and G4 structures in the cells (35). Originally discovered as a DNA repair enzyme, human APE1 plays a central role in the repair of endogenous oxidative and alkylating DNA damage through the well-conserved base excision repair (BER) pathway (36,37). Later, APE1 was independently identified as a multifunctional protein involved not only in DNA damage repair but also in regulating gene expression by its redox activity and is hence referred to as reduction–oxidation factor 1 (Ref-1) (38). APE1 reduces redox-sensitive cysteine residues in many oxidized TFs, including AP-1 (39), nuclear factor kappa B (40) and p53 (41), and increases their DNA binding by maintaining them in a reduced active state. Studies by us and others have also demonstrated that APE1 can act as a direct transcriptional co-activator or co-repressor of several different genes involved in cell growth, proliferation and chemotherapeutic drug resistance (42–46). APE1 is overexpressed in a variety of cancers, including pancreatic (47), prostate (48), cervical (49), gliomas (50), ovarian (51,52), lung (53) and colon (54). This increased expression has been associated with increased tumor growth, cell migration and drug resistance, as well as patients’ poor prognosis (50,51,55,56).

In this study, we demonstrate that APE1 is a G4-binding protein that plays a critical role in the formation of stable G4 structures in the genome and regulates *KRAS* expression in PDAC cells. Our study shows that recombinant APE1 binds to *KRAS* promoter G4 with high affinity *in vitro*. Loss of APE1 abrogates the formation of stable G4 structures and alters *KRAS* expression in PDAC cell lines. Knockdown (KD) of APE1 sensitizes PDAC cells to chemotherapy both *in vitro* and *in vivo*. Furthermore, we provide evidence that increased APE1 levels are associated more often with elevated levels of G4 DNA in patients with aggressive PDAC.

## MATERIALS AND METHODS

### Reagents and oligonucleotides

Primary antibodies used for the immunofluorescence studies were mouse monoclonal anti-APE1 (1:100; Novus Biologicals, Cat # NB100-116), rabbit polyclonal anti-APE1 (1:100) (57), mouse monoclonal anti-G4 clone 1H6 (1:50; Millipore Sigma, Cat # MABE1126) and anti-Acetyl Histone H3 (Lys27) antibody, clone 5E2.2 (1:100; Millipore Sigma, Cat # MABE670). Secondary antibodies used for the immunofluorescence studies were Alexa Fluor 594 goat anti-mouse IgG (H + L) (1:500; Life Technologies Corporation, Cat # A11005) or Alexa Fluor 488 goat anti-rabbit IgG (H + L) (1:500; Life Technologies Corporation, Cat # A11008). For the proximity ligation assay (PLA), the kit purchased was Duolink *In Situ* Red Starter Kit Mouse/Rabbit (Sigma-Aldrich, # DUO92101). Primary antibodies used in western blot studies include mouse monoclonal anti-APE1 (1:5000; Novus Biologicals, Cat # NB100-116) and mouse monoclonal anti-HSC70 (1:10 000; Santa Cruz Biotechnology, Cat # sc-7298). Primary antibodies used for the chromatin immunoprecipitation (ChIP) assay include mouse monoclonal anti-APE1 (Novus Biologicals, Cat # NB100-116), mouse monoclonal anti-G4 clone 1H6 (Millipore Sigma, Cat # MABE1126), mouse monoclonal IgG_2a_ anti-MAZ (133.7) (Santa Cruz Biotechnology, Cat # sc-130915) and mouse monoclonal IgG_2a_ anti-PARP1 (F-2) (Santa Cruz Biotechnology, Cat # sc-8007). Reagents used in the study include RNase A from bovine pancreas (Sigma, Cat # R4875-100MG), pyridostatin hydrochloride (PDS; Sigma, Cat # SML2690), doxycycline (Dox; Sigma, Cat # D9891), TMPyP4 (Millipore Sigma, Cat # 613560), gemcitabine hydrochloride (Millipore Sigma, Cat # G6423), oxaliplatin (Supelco, Cat # PHR1528) and 5-fluorouracil (5-FU; Sigma, Cat # F6627). All recombinant proteins used in the study were purified as described previously (58). All synthetic single-stranded DNA oligonucleotides, either unlabeled or 5′-6-carboxyfluorescein (6-FAM) labeled, were HPLC-purified grade and purchased from Integrated DNA Technologies in lyophilized form. They were dissolved in TE buffer to make a stock concentration of 100 μM and stored at −20°C. Oligonucleotide names and sequences are as follows: G4-forming KRAS oligo (5′-AGGGCGGTGTGGGAAGAGGGAAGAGGGGGAGG-3′) and non-G4-forming KRAS oligo (5′-AGTTCGGTGTGTTAAGAGTTAAGAGTTGGAGG-3′).

### Biological resources

The human embryonic kidney HEK-293T (ATCC, Cat # CRL-3216), mutant KRAS expressing pancreatic ductal adenocarcinoma PANC-1 (ATCC, Cat # CRL-3216) and MIA PaCa-2 (ATCC, Cat # CRM-CRL-1420) cell lines were maintained in high-glucose Dulbecco’s modified Eagle’s medium (DMEM; Thermo Fisher Scientific, Cat # 11965084) supplemented with 10% fetal bovine serum (FBS; Sigma, Cat # F2442) and an antibiotic mixture of 100 U/ml penicillin and 100 μg/ml streptomycin (Gibco-BRL). The wild-type (WT) KRAS expressing BxPC-3 (ATCC, Cat # CRL-1687) PDAC cell line was maintained in RPMI 1640 medium (1×) (Gibco, Cat # A10491-01) supplemented with 10% FBS and an antibiotic mixture of 100 U/ml penicillin and 100 μg/ml streptomycin. All cell lines were authenticated by STR DNA profiling by Genetica DNA Laboratories, Burlington, NC. For APE1 KD studies, PANC-1 and MIA PaCa-2 cell lines stably expressing APE1shRNA or non-targetable control (NTC) shRNA were maintained in 10% FBS supplemented DMEM with 1 μg/ml puromycin. To generate the Dox-inducible stable expression, APE1shRNA and NTCshRNA PANC-1 and MIA PaCa-2 cells, three different Dox-inducible human APE1shRNA constructs (shRNA #V3IHSHEG_5634292, #V3IHSHEG_6377584 and #V3IHSHEG_7228555 named as 1, 2 and 3, respectively; Dharmacon) and an NTCshRNA lentiviral SMARTvector construct with GFP were used. Lentiviral supernatants were generated by individually transfecting the shRNA lentiviral SMARTvector constructs into HEK-293T cells with packaging plasmids using X-tremeGENE HP DNA transfection reagent (Millipore Sigma, Cat # XTGHP-RO). Lentiviral supernatants were transduced into the aforementioned cell lines and selected with 1 μg/ml puromycin. All Dox-inducible APE1shRNA and NTCshRNA expressing stable cell lines were maintained in the respective media supplemented with tetracycline-free 10% FBS (Atlantic Biologicals) and 1 μg/ml puromycin. To generate stable APE1 KD in BxPC-3 cells, the APE1shRNA (5′-CAGAGAAATCTGCATTCTATTCTCGAGAATAGAATGCAGATTTCTCTG-3′) sequence was inserted in pLKO.1 puro (Addgene, plasmid # 8453) for transfection of the BxPC-3 cell line. Both the empty vector transfected and APE1shRNA transfected cell lines were maintained in RPMI medium supplemented with 10% FBS and an antibiotic mixture of 100 U/ml penicillin and 100 μg/ml streptomycin and containing 1 μg/ml puromycin. For rescue experiments, WT APE1 and redox mutant C65S/C99S APE1 cDNA were cloned into the empty vector pSF-CMV-NEO-COOH-3XFLAG (Sigma-Aldrich, Cat # OGS629).

### Immunofluorescence

Cells were cultured on coverslips (Fisherbrand, Cat # 12-542-B) and fixed in 4% paraformaldehyde (PFA; Sigma) in phosphate-buffered saline (PBS) for 15 min at room temperature (RT). Cells were subsequently permeabilized and blocked for 1 h at RT using a blocking buffer containing 0.5% Triton X-100 (Sigma), 10% goat serum (Thermo Fisher, Cat # 50062Z), glycine (Fisher BioReagents, Cat # BP381-1) and sodium azide in PBS. After permeabilization and blocking, cells were incubated in blocking buffer with primary antibodies overnight at 4°C and subsequently the corresponding secondary antibodies for 1 h at RT. Cells were then washed in PBS and mounted using mounting media with DAPI (Vector Laboratories, item # VV-93952-27). For G4 staining, an additional permeabilization step was performed after PFA fixation by incubating cells in PBS containing 0.5% Tween 20 (Fisher BioReagents, Cat # BP337-100) for 20 min at 37°C. Following Tween 20 permeabilization, cells were treated with 0.04 μg/μl RNase A and subsequently blocked and processed as described earlier. For the DNase experiments, cells were fixed in 4% PFA in PBS for 30 min, permeabilized with 0.2% Triton X-100 in PBS for 1 min and washed in PBS at RT. Cells were then incubated for 2 h at 37°C in DNase reaction buffer with or without 0.06 U/μl of DNase I (RQ1 DNase, Promega). The working DNase reaction buffer contained 40 mM Tris–HCl (pH 8), 5 mM CaCl_2_, 2 mM MgCl_2_ and 100 μg/ml bovine serum albumin. Following DNase digestion, the cells were washed in PBS and stained as described earlier. All immunofluorescence images were captured by confocal microscopy or super-resolution (∼100 nm) structured illumination microscopy (SIM), where indicated. Confocal microscope images were captured using the Zeiss LSM 800 with Airyscan microscope in the Advanced Microscopy Core Facility (AMCF) at the University of Nebraska Medical Center (UNMC). Three-dimensional SIM images were collected with the Zeiss ELYRA PS.1 super-resolution microscope in the AMCF at UNMC. Pearson’s correlation coefficients were calculated using the ImageJ software JACoP colocalization analysis module. Quantification of colocalization was determined by establishing a threshold using the JACoP threshold optimizer followed by calculation of correlation coefficients.

### Proximity ligation assay

Cells were cultured on coverslips and fixed in 4% PFA in PBS for 15 min at RT. An additional permeabilization step was performed after PFA fixation by incubating the cells in PBS containing 0.5% Tween 20 for 20 min at 37°C. Following Tween 20 permeabilization, cells were treated with 0.04 μg/μl RNase A and subsequently blocked using the Duolink blocking solution. The PLA was performed following the manufacturer’s protocol for Duolink *In Situ* Red Starter Kit Mouse/Rabbit (Sigma-Aldrich, # DUO92101). The primary antibodies used for the PLA studies were rabbit polyclonal anti-APE1 (1:100) and anti-G4 clone 1H6 (1:50). Confocal images were captured using the Zeiss LSM 800 with Airyscan microscope in the AMCF at UNMC. For quantification, the number of foci per cell was counted for 50 cells under each experimental condition using the ImageJ software.

### Western blot

Whole cell extracts were prepared, and western blot was performed as we have described in our previous study (59).

### Fluorescence polarization assay

The fluorescence polarization (FP) measurements were obtained in a SpectraMax M5 Multimode Microplate Reader using SoftMax Pro software. 6-FAM-labeled G4-forming KRAS oligonucleotide (oligo) at a concentration of 10 nM was incubated in a buffer containing 50 mM Tris–HCl (pH 7.5), 1 mM MgCl_2_, 1 mM DTT, 0.1 mM EDTA and 50 mM KCl alone or with increasing doses of purified recombinant proteins (1.25–10 240 nM), G4 ligands, 1H6 antibody or unlabeled oligo in triplicates for 30 min at RT in the dark in a black opaque Perkin Elmer 384-well plate. The total volume of the samples in each well was 25 μl, and each experiment with triplicate samples was repeated three times. The fluorescence (RFUs), FP and anisotropy values were recorded at 25°C with the excitation filter set at 493 nm and emission filter set at 525 nm with a cutoff of 515 nm. The FP values expressed in the units of millipolarization versus the concentration of the proteins, ligands, antibodies or oligo were plotted using the nonlinear regression (curve fit) option in Prism 8.0, and dissociation constants (*K*_d_) and EC_50_ values were obtained.

### Tryptophan fluorescence quenching

The fluorescence measurements were obtained using a Varian Cary Eclipse Fluorescence Spectrophotometer (Walnut Creek, CA). A working concentration (500 nM) of WT APE1 protein was prepared in a buffer containing 50 mM Tris–HCl (pH 7.5), 1 mM MgCl_2_, 1 mM DTT, 0.1 mM EDTA and 50 mM KCl. The fluorescence emission spectrum was obtained for the free protein from 300 to 450 nm, using an excitation maximum of 280 nm, with the emission and excitation slits set at 5 nm. This protein solution was titrated with increasing doses of G4-forming KRAS oligo (0 nM to 1 μM) and for each concentration of the oligo, the emission spectrum was recorded. The spectra are reported as fluorescence intensity [arbitrary unit (a.u.)] versus wavelength (nm).

### Circular dichroism spectroscopy

Circular dichroism (CD) spectra were obtained on a JASCO J-810 Spectropolarimeter, equipped with a Peltier thermoelectric type temperature control system and flow-through HPLC cell. The instrument was controlled by Jasco’s Spectra Manager™ software. The experiments were carried out with 1 μM oligonucleotide solutions in 50 mM Tris–HCl (pH 7.5), 2 mM MgCl_2_ and 1 mM DTT alone or in combination with 1 μM of the recombinant proteins. The spectra were recorded in a 10 mm quartz cuvette at 20°C. Scans were performed over a wavelength range of 210–330 nm with a response time of 0.5 s, 1 nm pitch and 1 nm bandwidth. Blank spectra of samples containing buffer were subtracted from that of DNA samples. Each spectrum was recorded five times, smoothed and subtracted to the baseline. The spectra are reported as ellipticity (measured in units of millidegrees) versus wavelength (nm).

### UV–Vis absorption titration experiment

Absorption spectra were measured on a thermoelectrically controlled Aviv 14 DS UV/Vis Spectrophotometer (Lakewood, NJ) in a 1 cm path length quartz cuvette. The reference solution contained 10 mM Tris–HCl (pH 7.5), 1 mM EDTA and 50 mM KCl. UV–Vis absorption titrations were carried out by the stepwise addition of unlabeled G4-forming KRAS oligo (0 nM to 1.8 μM) solution to the quartz cuvette containing 4 μM solution of TMPyP4 in the above-mentioned buffer. Absorption spectra were recorded in the 350–500 nm wavelength range at RT. The titration was terminated when the wavelength and intensity of the absorption band for TMPyP4 did not change anymore upon three successive additions of the G4 oligo-containing solution. The spectra are reported as absorbance (a.u.) versus wavelength (nm).

### Chromatin immunoprecipitation

Cells were plated in 150 mm cell culture dishes. The following day, cells were incubated with 1% formaldehyde in PBS for 15 min at RT to cross-link protein–DNA complexes and subsequently washed with PBS. Cells were collected in PBS containing 1× protease inhibitor cocktail (Roche cOmplete™) with a plastic cell scraper and pelleted at 1000 rpm for 10 min at 4°C. The pelleted cells were lysed and incubated on ice in SDS lysis buffer (1% SDS, 10 mM EDTA, 50 mM Tris–HCl, pH 8) for 10 min and subsequently subjected to sonication (Misonix Sonicator 3000). Sonication was performed on ice with eight rounds of 15 s pulses with 15 s pause between each pulse. The sonicated lysate was centrifuged at 14 000 rpm for 15 min at 4°C, and the supernatant containing the clear sheared chromatin lysate was collected. The collected lysate was diluted 1:10 to a total volume of 2 ml with ChIP dilution buffer containing 0.01% SDS, 1.1% Triton X-100, 1.2 mM EDTA, 16.7 mM Tris–HCl (pH 8) and 167 mM NaCl. Immunoprecipitation was performed by incubating 5 μg of the respective antibody with the diluted lysate overnight at 4°C with constant nutating rocking. The next day, 25 μl of Protein A/G Dynabeads™ (Invitrogen) was added to each reaction and incubated for 2 h at 4°C with constant nutating rocking. The immunoprecipitates were washed sequentially with low-salt immune complex wash buffer (0.1% SDS, 1% Triton X-100, 2 mM EDTA, 20 mM Tris–HCl, pH 8, 150 mM NaCl), high-salt immune complex wash buffer (0.1% SDS, 1% Triton X-100, 2 mM EDTA, 20 mM Tris–HCl, pH 8, 500 mM NaCl), LiCl immune complex wash buffer (0.25 M LiCl, 1% NP-40, 1% sodium deoxycholate, 1 mM EDTA, 10 mM Tris–HCl, pH 8) and TE buffer (10 mM Tris–HCl, 1 mM EDTA, pH 8). The protein–DNA complexes were eluted in ChIP elution buffer (1% SDS, 0.1 M NaHCO_3_) and de-cross-linked in 200 mM NaCl overnight at 65°C. ChIP DNA was purified by RNase treatment, proteinase K digestion, phenol–chloroform extraction and precipitation by 100% ethanol using a standard protocol. The ChIP-purified DNA was finally dissolved in ultrapure DNase/RNase-free water and was used for subsequent assays.

### ChIP-qPCR analysis

The ChIP DNA and 1% input DNA were subjected to SYBR Green-based real-time PCR (7500 Real-Time PCR System; Applied Biosystems) with primers for KRAS G4 (forward: 5′-GTACGCCCGTCTGAAGAAGA-3′; reverse: 5′-GAGCACACCGATGAGTTCGG-3′) and Ctr-2 (forward: 5′-CTCCGACTCTCAGGCTCAAG-3′; reverse: 5′-CAGCACTTTGGGAGGCTTAG-3′). Data were represented as % input calculation [2^(adjusted input − ChIP CT) × 100].

### Quantitative RT-PCR

For quantitative real-time PCR (qRT-PCR), total RNA was isolated from cells using the TRIzol method. cDNA synthesis from 1 μg of total RNA was performed with the MulV RT kit (Invitrogen) using random hexamer primers. For qRT-PCR analyses, 1/50th of each reaction was used. Analysis was performed using SYBR Green (Applied Biosystems) for detection with an Applied Biosystems StepOne Plus System. Fold change was calculated by specific gene 2^−(target − reference)/*GAPDH* 2^−(target − reference). The gene-specific primers used were as follows: KRAS (forward: 5′-TCTTGCCTCCCTACCTTCCACAT-3′; reverse: 5′-CTGTCAGATTCTCTTGAGCCCTG-3′) and GAPDH (forward: 5′-TGGGCTACACTGGAGCACCAG-3′; reverse: 5′-GGGTGTCGCTGTTGAAGTCA-3′).

### Colony formation assay

Five hundred cells were seeded in each well of six-well plates and incubated at ambient conditions in appropriate medium for 24 h to allow for their attachment. Following this incubation, cells were either left untreated (control group) or treated with increasing doses of oxaliplatin (0.5–8 μM), 5-FU (2–10 μM) or gemcitabine (0.005–0.5 μM) for 24 h. After 24 h, the media containing drugs were discarded and fresh media were added, and the cells were allowed to grow for 12 days under ambient cell culture conditions. Following this, the media were removed and the colonies in the wells were stained with a 0.5% crystal violet solution (made in 25% methanol and stored at RT) for 10 min. Subsequently, the plates were carefully rinsed in double-distilled water. The plates were allowed to dry, and the number of colonies was counted and plotted as percent cell viability against the drug concentrations. For each condition, three independent experiments were done in triplicates.

### Xenograft studies

All animal experiments were performed with the approval of the UNMC Institutional Animal Care and Use Committee. The experiments and reports adhered to the Animal Research: Reporting of *In Vivo* Experiments guidelines. MIA PaCa-2^APE1shRNA^ cells (2 × 10^6^ in 100 μl medium with Matrigel) were injected subcutaneously over the left and the right flanks in 6-week-old male athymic nude mice (Charles River). The average weight was 27 ± 3.6 g. Dox (2 mg/ml) was given in the drinking water with 1% sucrose to the Dox-treated group. All the water bottles were changed every 2 days. Subcutaneous tumors were allowed to grow for 1–2 weeks before treatments. The mice were divided into six treatment groups (*n* = 5 in each group) and received treatments twice per week for 4 weeks. The drugs 5-FU 30 mg/kg and oxaliplatin 2 mg/kg were injected intraperitoneally. Normal saline (100 μl) was given to the vehicle control group. Body weights and tumor volumes were measured before each treatment. The mice were euthanized in a gas canister with gradual fill carbon dioxide at the end of the treatment cycles. Xenograft tumors were fixed in formalin, and paraffin-embedded tissue sections were used to perform immunohistochemical (IHC) staining with anti-APE1 antibody (1:600).

### Patient tissue sample studies and immunohistochemical analysis

PDAC patient tissue microarrays (TMAs) were obtained from the Rapid Autopsy Program for Pancreas at UNMC. Tissues were collected in accordance with the institution’s review board approval and informed consent waiver. The deparaffinized sections were stained as per standard IHC protocol. The antibodies used were anti-APE1 (1:600) and anti-G4 clone 1H6 (1:200). IHC staining scores for APE1 and G4 were calculated by multiplying scores for extent of staining (percentage of stained cells) and intensity of staining. The criteria used to score the staining of the tissue samples were based on the percentage of cells stained: 0% stained cells, score 0; 1–10% stained cells, score 1; 11–50% stained cells, score 2; 51–80% stained cells, score 3; and 81–100% stained cells, score 4. The criteria used to score the tissue sample staining based on intensity were as follows: no staining, score 0; weak staining intensity, score 1; moderate staining intensity, score 2; and high staining intensity, score 3. The information regarding the cancer stage, response to treatment and survival days for each patient was obtained from the Rapid Autopsy Program for Pancreas database.

## RESULTS

### APE1 plays a crucial role in the formation of stable G4 structures in PDAC cell lines

**Figure 1.**
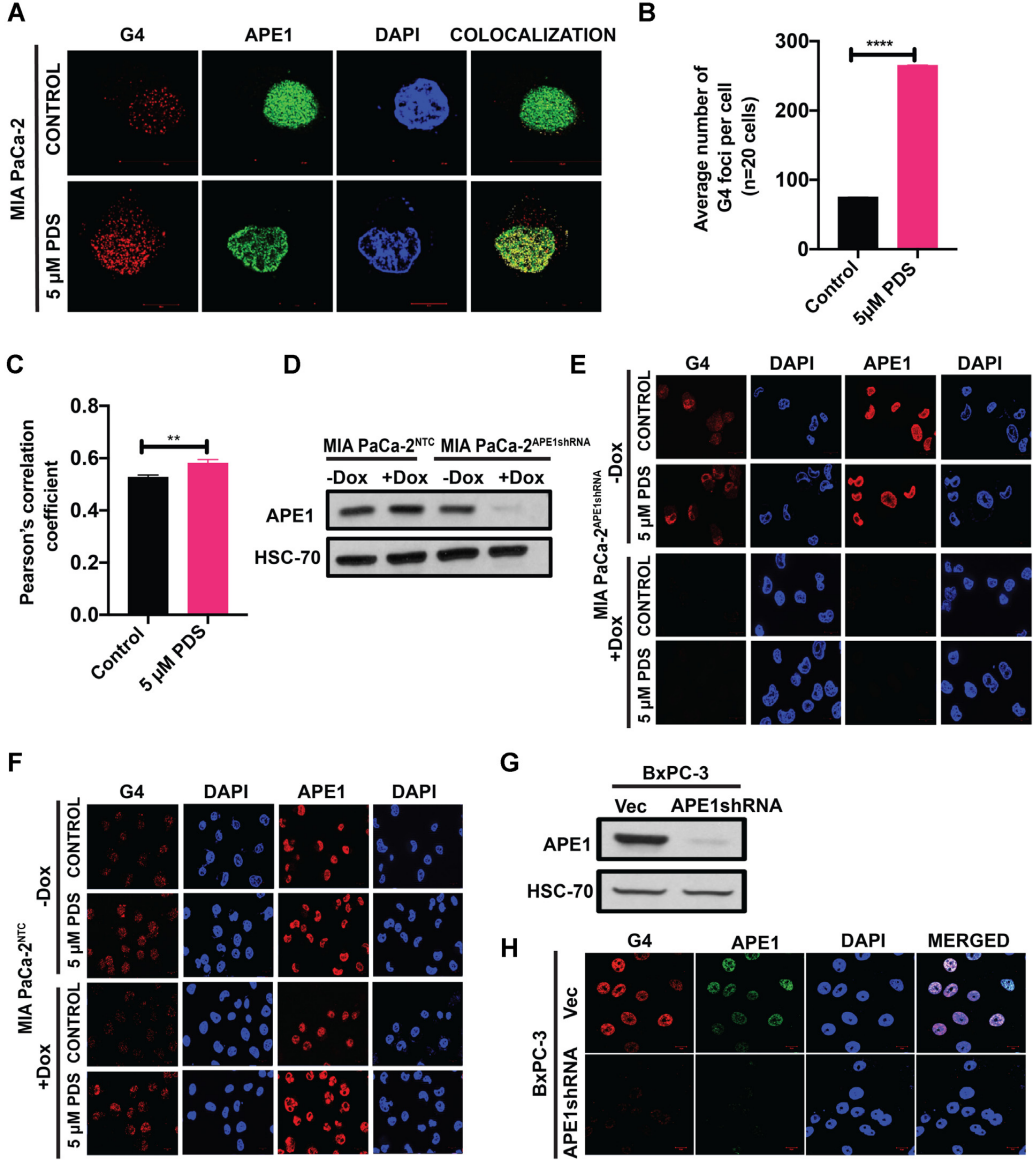
APE1 plays a crucial role in the formation of G4 structures in cells. **(A)** SIM images of MIA PaCa-2 cells immunostained with α-1H6 and α-APE1 antibodies before or after treatment with PDS. Cells were counterstained with DAPI (magnification: 63×). **(B)** Quantification of the average number of G4 per cell (*n* = 20 cells). An unpaired Student’s *t*-test comparing control versus 5 μM PDS-treated samples was used to determine the *P-*values (****P< 0.0001). Error bars denote ±SD. **(C)** Pearson’s correlation coefficient was calculated (*n* = 20 cells) as a measure of colocalization frequency. An unpaired Student’s *t*-test comparing control versus PDS-treated samples was used to determine the *P-*value (**P< 0.01). Error bars denote ±SD. **(D)** MIA PaCa-2 cells expressing NTCshRNA (MIA PaCa-2^NTCshRNA^) or APE1shRNA (MIA PaCa-2^APE1shRNA^) under a Dox-inducible promoter were treated without or with 2 μg/ml Dox for 4 days and APE1 levels were examined by western blot using α-APE1 and α-HSC70 (loading control) antibodies. **(E)** MIA PaCa-2^APE1shRNA^ cells treated without or with Dox were immunostained with α-1H6 and α-APE1 antibodies and visualized by confocal microscopy (magnification: 63×; scale bars: 20 μm). **(F)** MIA PaCa-2^NTC^ cells treated without or with Dox were immunostained with α-1H6 and α-APE1 antibodies and visualized by confocal microscopy (magnification: 63×; scale bars: 10 μm). **(G)** APE1 levels were examined by western blot using α-APE1 and α-HSC70 (loading control) antibodies in BxPC-3 cells stably expressing empty vector and APE1shRNA. **(H)** BxPC-3 cells stably expressing empty vector and APE1shRNA were immunostained with α-1H6 and α-APE1 antibodies and visualized by confocal microscopy (magnification: 63×; scale bars: 10 μm). For each immunofluorescence-based study, three independent experiments were performed.

We examined the formation of G4 structures in PDAC cell lines MIA PaCa-2 and PANC-1 using the G4 structure-specific antibody, clone 1H6. This antibody has been extensively used for analyzing the G4 structures in cells in several earlier studies (60,61). Moreover, the FP assay revealed that 1H6 antibody has high affinity for binding (*K*_d_ = 0.5 ± 0.3 nM) to a preformed *KRAS* promoter G4 structure *in vitro* (Supplementary Figure S1A). Using this antibody and SIM, we could detect G4 foci primarily in the nuclei of MIA PaCa-2 cells (Figure 1A). This immunostaining of G4 DNA in cells was sensitive to DNase but not to RNase A treatment, confirming the specificity of the antibody toward the DNA G4 structures (Supplementary Figure S1B). Using confocal microscopy, similar results for G4 staining were obtained in PANC-1 cell line (Supplementary Figure S1C). We observed colocalization between G4 and APE1 staining in these cells (Figure 1A and Supplementary Figure S1C). Treatment with G4-stabilizing ligand PDS (62,63) increased the number of G4 foci and colocalization between G4 foci and APE1 staining (Figure 1A–C). On the other hand, downregulation of APE1 by stable expression of APE1shRNA under a Dox-inducible promoter (Figure 1D and Supplementary Figure S1D) led to a significant reduction in the number of detectable G4 foci in MIA PaCa-2 (Figure 1E) and PANC-1 (Supplementary Figure S1E) cell lines. Furthermore, treatment with H_2_O_2_ and PDS showed no effect on the G4 staining in APE1 downregulated cells (Figure 1E and Supplementary Figure S1E). In contrast, Dox treatment in cells stably expressing NTCshRNA had no effect on the G4 staining (Figure 1F). We also examined the effect of APE1 KD on G4 staining in the WT *KRAS* expressing BxPC-3 cell line. We observed an appreciable number of G4 foci and APE1 staining in BxPC-3 cells expressing control empty vector (Figure 1H, upper panel). KD of APE1 in BxPC-3 cells using stable expression of APE1shRNA (Figure 1G) abrogated the G4 staining in these cells (Figure 1H, lower panel).

**Figure 2.**
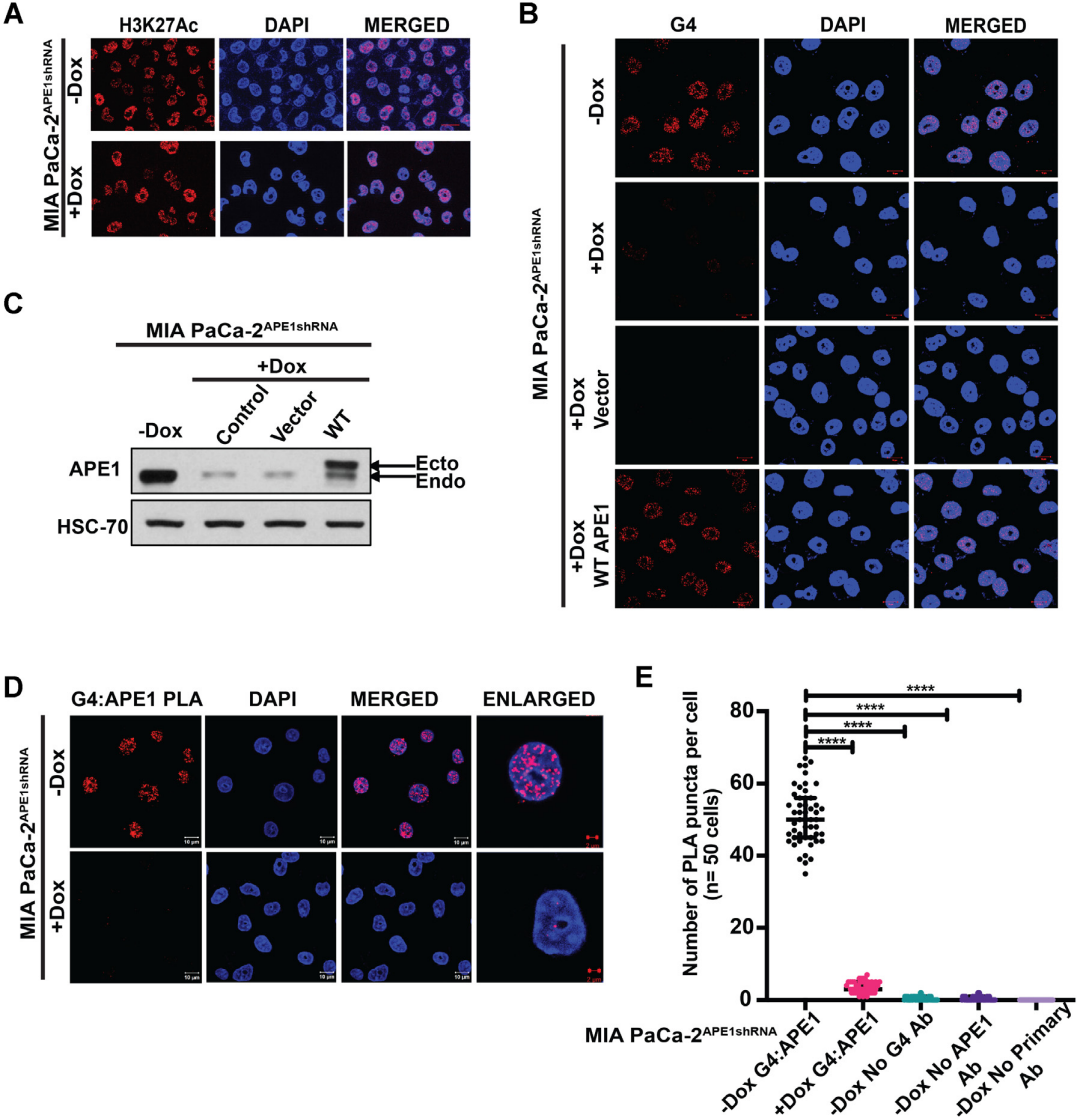
APE1 interacts with G4 structures and APE1 KD abrogates G4 staining in cells. **(A)** MIA PaCa-2^APE1shRNA^ cells treated without (top) or with (bottom) Dox were immunostained with α-H3K27Ac antibody and visualized by confocal microscopy (magnification: 63×; scale bars: 10 μm). **(B)** APE1 levels were downregulated in MIA PaCa-2^APE1shRNA^ using Dox and these cells were then transfected with vector control or plasmid expressing 3× FLAG-WT APE1. Twenty-four hours after transfection, cells were immunostained with α-1H6 antibody and visualized by confocal microscopy (magnification: 63×; scale bars: 10 μm). **(C)** APE1 levels in these cell extracts were examined by western blot analysis with α-APE1 and α-HSC70 (loading control) antibodies. **(D)** MIA PaCa-2^APE1shRNA^ cells were treated without (top) or with (bottom) Dox and PLA was performed with antibodies against APE1, G4 and PLA probes. G4:APE1 PLA foci were visualized by confocal microscopy images (magnification: 63×; scale bars: 10 μm; enlarged scale bars: 2 μm). **(E)** Quantitation of the number of PLA puncta per cell (*n* = 50 cells). An unpaired Student’s *t*-test comparing the number of PLA puncta in control versus APE1 KD and control versus antibody control samples was used to determine the *P-*value (****P< 0.0001). Three independent experiments were performed.

To eliminate the possibility that APE1 downregulation can affect the staining of other chromatin-bound proteins in general, we immunostained the APE1 downregulated MIA PaCa-2 and PANC-1 cells with histone H3K27Ac antibody. We observed no change in the staining of H3K27Ac under APE1 KD conditions, indicating that downregulation of APE1 does not affect staining of other chromatin-bound protein, but it specifically reduced G4 staining in the PDAC cell lines (Figure 2A and Supplementary Figure S2A). Re-introduction of WT APE1 expressing plasmid but not empty vector plasmid in APE1 KD cells was able to rescue G4 foci formation, confirming the requirement of APE1 for stable G4 formation in the genome (Figure 2B and C). The association of APE1 with G4 structures in cells was also confirmed by PLA using control and APE1 KD cells (Figure 2D). While a significant number of APE1:G4 PLA foci were seen in control cells, APE1 KD significantly reduced the number of PLA foci (Figure 2D and E). As controls, we used only APE1 antibody, only G4 antibody and no primary antibody, all of which gave us almost no foci, emphasizing the specificity of the assay (Supplementary Figure S2B and Figure 2E). Together, our results suggest that APE1 colocalizes with G4 in PDAC cells and plays a critical role in the formation of G4 structures in the genome.

### APE1 binds to *KRAS* promoter G4 structure with high affinity

**Figure 3.**
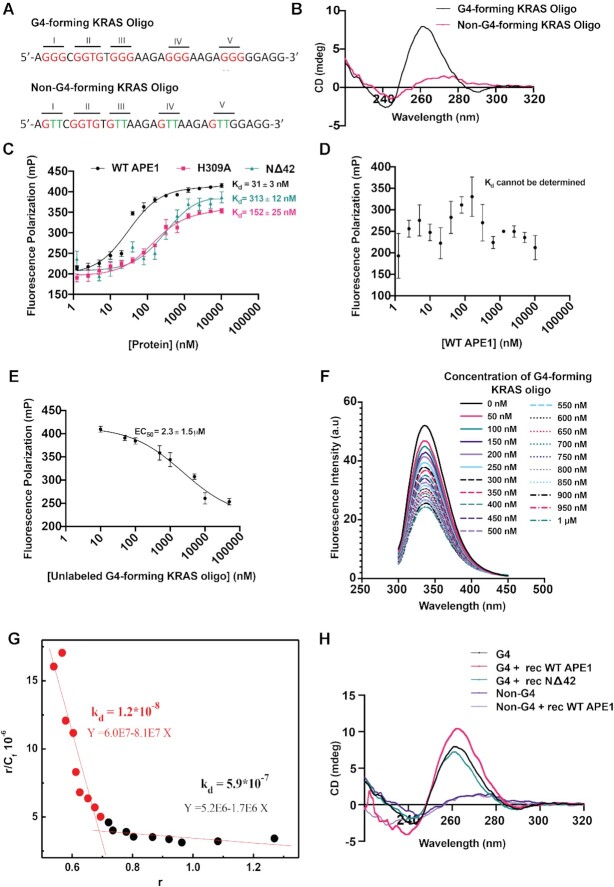
APE1 binds with strong affinity to *KRAS* promoter G4 structure. **(A)** The sequence of 32R (G4-forming KRAS oligo), a 32-mer *KRAS* promoter sequence containing the G4-forming motif and a 32-mer sequence in which G residues were mutated to T (shown in green; non-G4-forming KRAS oligo) are shown. **(B)** CD spectra of G4-forming KRAS oligo and non-G4-forming KRAS oligo at 20°C in the presence of 50 mM KCl. The *Y*-axis indicates the ellipticity signal expressed in millidegrees. **(C)** 6-FAM-labeled G4-forming KRAS oligo (10 nM) was incubated with increasing concentrations of WT (black), or H309A (pink) or NΔ42 (teal) APE1 recombinant proteins in a buffer containing 50 mM KCl, 50 mM Tris–HCl (pH 7.5), 1 mM MgCl_2_, 1 mM DTT and 0.1 mM EDTA, and FP values were recorded. The dissociation constant (*K*_d_) was calculated [nonlinear regression (curve fit) total saturation binding on Prism 8.0] from three independent experiments with triplicate samples; average ± SD *K*_d_ values are shown on the graph. **(D)** FP assay was performed with 6-FAM-labeled non-G4-forming KRAS oligo (10 nM) incubated with increasing concentrations of WT APE1 protein. **(E)** 6-FAM-labeled G4-forming KRAS oligo (10 nM) was incubated with a saturating dose (640 nM) of WT APE1 protein and then titrated with increasing concentrations of unlabeled G4-forming KRAS oligo, and FP values were plotted using Prism 8.0. The EC_50_ was calculated from three independent experiments performed with triplicate samples. **(F)** Titration for intrinsic fluorescence of WT APE1 with G4-forming KRAS oligo. The titration was carried out in a 500 μl quartz cuvette containing a solution of WT APE1 (500 nM) in a buffer containing 50 mM Tris–HCl (pH 7.5), 1 mM MgCl_2_, 1 mM DTT, 0.1 mM EDTA and 50 mM KCl. Increasing doses (0 nM to 1 μM) of G4-forming KRAS oligo were added to the WT APE1 solution and for each concentration of the oligonucleotide the fluorescence spectra were recorded at 20°C between 300 and 450 nm with excitation wavelength of 280 nm after 10 min of incubation. The spectra are reported as fluorescence intensity (a.u.) versus wavelength (nm). Three independent experiments were performed. **(G)** Scatchard plot showing the dissociation constant (*K*_d_) for specific (red) and nonspecific (black) binding of WT APE1 protein to the *KRAS* G4 structure. **(H)** CD spectra of G4-forming KRAS alone or in combination with WT APE1 protein or NΔ42 protein and non-G4-forming KRAS oligo alone or in combination with WT APE1 protein at 20°C in the presence of 50 mM KCl. The *Y*-axis indicates the ellipticity signal expressed in millidegrees.

The observations that APE1 colocalizes with G4 structures and is required for the formation of G4 structures in PDAC cells led us to test whether APE1 binds to G4 structures in cells. We first examined the ability of recombinant WT APE1 to bind directly to a preformed *KRAS* promoter G4 structure using *in vitro* biophysical assays. The *KRAS* promoter contains three G4 motifs, among which the proximal G4 motif (−148 and −116 bp), otherwise known as 32R, has been well characterized to form G4 *in vitro* and shown to be functionally involved in regulating *KRAS* expression in cells (30). We synthesized a 6-FAM-labeled 32-mer oligonucleotide corresponding to the *KRAS* G4-proximal motif (which we named as the G4-forming KRAS oligo) (Figure 3A), and induced G4 folding by incubating the oligo in the presence of 50 mM KCl. We confirmed the formation of a parallel G4 structure by a CD spectroscopy assay that demonstrated a strong positive ellipticity at around 265 nm and a weak negative ellipticity at around 240 nm, which are the established CD signatures for a parallel G4 structure (Figure 3B). As a negative control, we used a KRAS oligo in which several G residues were replaced with T so that it cannot form G4 structure (non-G4-forming KRAS oligo) (Figure 3A). We confirmed the inability of the non-G4-forming KRAS oligo to form parallel G4 structure by CD spectroscopy where the characteristic CD signature for parallel G4 structure was absent (Figure 3B). The FP assay was performed using the G4-forming KRAS oligo and increasing doses of purified recombinant WT APE1 protein. We found that the WT APE1 protein has a very strong affinity for binding to the *KRAS* G4 structure, with a dissociation constant (*K*_d_) of 31 ± 3 nM, indicating that APE1 can bind with high affinity to a G4 structure without any AP site damage (Figure 3C). In contrast, WT APE1 did not show any measurable binding affinity toward the non-G4-forming KRAS oligo that cannot form G4 structure, thus confirming that APE1 has a high affinity toward a G4 structure (Figure 3D). A competition assay with unlabeled G4-forming KRAS oligo revealed an EC_50_ value of 2.3 ± 1.5 μM, further emphasizing the high affinity binding between APE1 and *KRAS* G4 structure (Figure 3E). We also independently confirmed APE1’s binding to the G4-forming KRAS oligo by a tryptophan (Trp) fluorescence quenching assay. APE1 has seven Trp residues whose intrinsic fluorescence is highly sensitive to changes in the local environment upon addition of a binding partner (64). We determined the binding of WT APE1 to *KRAS* G4 structure by measuring the quenching of Trp fluorescence upon addition of increasing doses of preformed *KRAS* G4 to a solution of WT APE1 protein (Figure 3F). We calculated the concentration of the bound protein (*C*_b_), the concentration of the free protein (*C*_f_) and *r*, which is the ratio of *C*_b_ to the concentration of G4-forming KRAS oligo in the solution. A Scatchard plot revealed a *K*_d_ of 12 nM, indicating a very strong binding affinity of APE1 to G4 (Figure 3G). Taken together, data from both our FP assay and the Trp fluorescence quenching studies suggest that WT APE1 protein has very strong binding affinity to *KRAS* promoter G4 structure, with a dissociation constant in the nanomolar range.

To investigate which domain of APE1 is involved in its strong binding to *KRAS* promoter G4 structure, we performed FP assays with APE1 catalytically inactive mutant H309A, N-terminal 42-amino acid deletion mutant NΔ42 and preformed *KRAS* G4 structure. His309 residue, in the active site pocket of APE1, is necessary for the catalytic activity (AP-endonuclease) of APE1 (65). The H309A mutant showed ∼5-fold less affinity (*K*_d_ = 152 ± 25 nM) for *KRAS* promoter G4 compared to WT APE1 protein (Figure 3C). Previous studies have shown that the highly positively charged and unstructured N-terminal domain of APE1 participates in its interactions with DNA or RNA (66,67). To determine whether the N-terminus of APE1 is required for its strong binding to the *KRAS* G4 structure, we performed the FP assay with an N-terminal deletion mutant NΔ42. The FP assay revealed a *K*_d_ of 313 ± 12 nM, which is ∼10-fold higher than that for WT APE1 protein, indicating that the N-terminus contributes to the binding with the *KRAS* G4 structure (Figure 3C). To determine whether the N-terminus 1–42 amino acids of APE1 are sufficient to bind to the *KRAS* G4 structure, we purified GST-tagged APE1 1–42-amino acid peptide and examined its binding to the *KRAS* G4 structure using the FP assay. We found that this peptide by itself possesses no measurable binding affinity to the *KRAS* G4 structure (Supplementary Figure S3). These data together suggest that even though the positively charged N-terminal domain contributes to G4 binding in the context of the full-length APE1, the other domains of the protein also play an important role in its strong binding to the G4 structure *in vitro*.

Several earlier studies demonstrated that *KRAS* proximal G4 oligonucleotide is able to form a parallel G4 structure in the presence of 50 mM KCl (68). Consistent with this, we also observed formation of *KRAS* parallel G4 when the oligo was incubated in the presence of KCl *in vitro* (Figure 3B). Interestingly, addition of recombinant WT APE1 to the G4-forming KRAS oligo resulted in an increase in positive ellipticity at around 265 nm, indicating that WT APE1 has the ability to bind and promote the folding/stacking of G4 structure (Figure 3H). As a negative control, we used non-G4-forming KRAS oligo, to which addition of recombinant WT APE1 had no effect as indicated by no change in the CD spectra signature (Figure 3H). We also performed CD spectroscopy with the NΔ42 mutant protein and observed almost no effect on the ellipticity at 265 nm with the G4-forming KRAS oligo indicating the absence of the stacking effect (Figure 3H). This suggests that the positively charged N-terminal domain of APE1 may facilitate the stacking of the G-tetrads to promote G4 folding *in vitro*.

### APE1 is associated with *KRAS* promoter G4 region in cells and modulates *KRAS* expression

**Figure 4.**
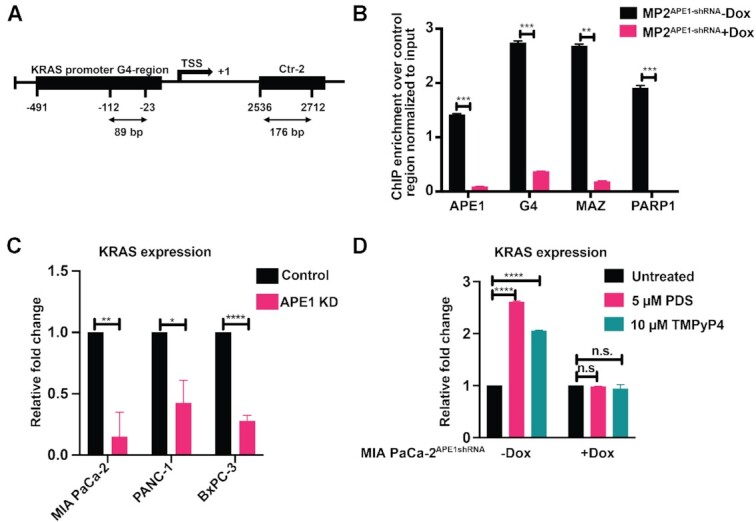
APE1 is associated with *KRAS* promoter G4 region in cells and regulates *KRAS* expression. **(A)** Schematic representation of G4 and non-G4 (Ctr-2) regions in *KRAS* promoter with respect to TSS. The length of each amplified DNA fragment for the real-time ChIP-PCR experiments is indicated. **(B)** MIA PaCa-2^APE1shRNA^ cells were treated without and with Dox and ChIP assays were performed using antibodies against APE1, G4, MAZ and PARP-1. Enrichment on *KRAS* promoter G4 region (−23 to −112) and a non-G4 control region (Ctr-2; +2536 to +2712) was examined to analyze the occupancy of APE1, G4, MAZ and PARP1 in the G4 region over the control region by real-time ChIP-PCR. Relative fold changes normalized to input controls are shown. **(C)** Relative *KRAS* gene expression (normalized to GAPDH) was measured by real-time PCR for control and APE1 KD MIA PaCa-2, PANC-1 and BxPC-3 cells. **(D)** qRT-PCR assays were performed with MIA PaCa-2^APE1shRNA^ cells that were treated without and with Dox and then with or without 5 μM PDS or 10 μM TMPyP4 for 2 h, and relative *KRAS* gene expression (normalized to GAPDH) was calculated. The *P-*values were determined using an unpaired Student’s *t*-test (****P< 0.0001, ***P< 0.001, **P< 0.01, *P< 0.05, n.s. (nonsignificant) = *P*> 0.05). Error bars denote ±SD. Three independent experiments were performed in triplicates.

The strong binding affinity of WT APE1 to *KRAS* G4 structure *in vitro* and their colocalization and interaction in cells led us to examine the occupancy of APE1 on the *KRAS* promoter G4 region. We tested for the co-occupancy of APE1 and G4 in the *KRAS* proximal promoter region by ChIP using APE1 and G4-specific antibody followed by a promoter-directed quantitative PCR (ChIP-qPCR) assay. Our results demonstrate significant enrichment of APE1 and G4 structures in the *KRAS* promoter G4 region as opposed to the control non-G4 sequence-containing region (Ctr-2) (Figure 4A and B). To test whether APE1 is required for stable G4 formation on *KRAS* promoter in cells, we used control and APE1 KD MIA PaCa-2 cells. Promoter-directed G4-ChIP-qPCR analysis revealed a significant reduction in G4 enrichment on *KRAS* promoter upon APE1 KD (Figure 4B). Several earlier studies have shown that the recruitment of TFs MAZ and PARP1 to the *KRAS* promoter G4 region activates *KRAS* expression (31,69). Our promoter-directed ChIP-qPCR assay revealed that APE1 KD significantly reduced the enrichment of PARP1 and MAZ on the *KRAS* promoter G4 region compared to the control region (Figure 4B). This suggests that APE1 plays a crucial role in PARP1 and MAZ TF loading onto the *KRAS* G4 promoter region.

Next, we examined the effect of APE1 KD on *KRAS* expression. Using qRT-PCR analysis, we demonstrated that downregulation of APE1 by stable expression of APE1shRNA in MIA PaCa-2, PANC-1 and BxPC-3 cell lines significantly decreased *KRAS* expression (Figure 4C). We also examined the effect of G4-stabilizing ligands, PDS and TMPyP4, on *KRAS* expression in control and APE1 KD PDAC cell lines. First, we confirmed the robust binding of these G4 ligands to *KRAS* G4 by the FP assay (Supplementary Figure S4A and B). Since these ligands have an extended aromatic core that can change the FP by binding to the fluorophore (6-FAM) tag of the oligo, but not by directly binding to the G4 structure, we performed a UV–Vis titration experiment with TMPyP4 and unlabeled G4-forming KRAS oligo (Supplementary Figure S4C). We observed a dissociation constant (*K*_d_) of 11 nM between TMPyP4 and *KRAS* G4, thus confirming that the ligand indeed has a very strong affinity to bind to the G4 structure (Supplementary Figure S4D). Upon treatment with PDS or TMPyP4, we observed a significantly increased *KRAS* expression in MIA PaCa-2 cells (Figure 4D). In contrast, the same treatment in the APE1 KD cells failed to significantly increase *KRAS* expression (Figure 4D), again highlighting the critical role of APE1 in modulating the expression of *KRAS* through regulation of the formation of stable G4 structure. Similar changes in *KRAS* expression were observed upon treating the PANC-1 cell line with PDS (Supplementary Figure S4E). Taken together, these data demonstrate that APE1 plays a crucial role in G4 structure formation in the *KRAS* promoter, thereby regulating *KRAS* expression.

### Redox function of APE1 is not involved in the regulation of G4 structure formation and the G4-mediated *KRAS* gene expression

**Figure 5.**
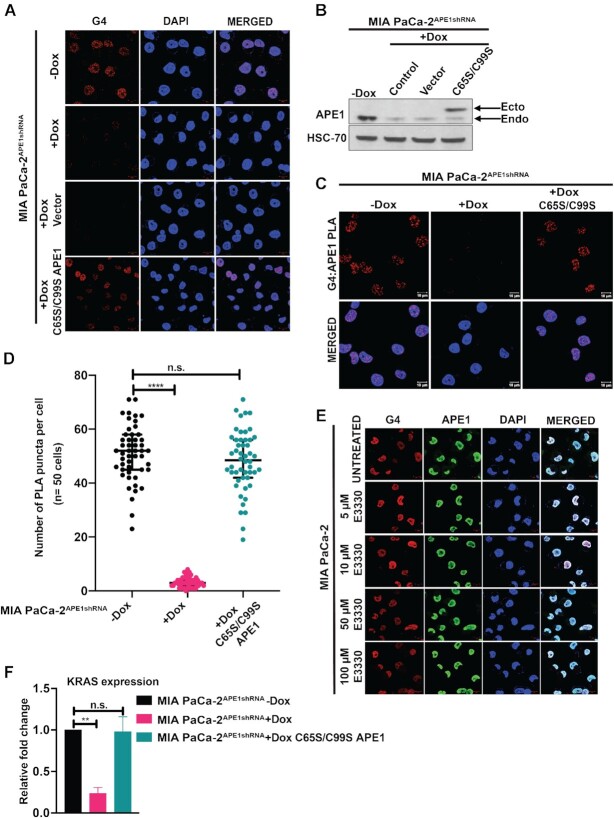
Redox function of APE1 is not involved in the regulation of G4 structure formation and G4-mediated *KRAS* expression. **(A)** MIA PaCa-2^APE1shRNA^ cells were treated with Dox and then transfected with 3× FLAG-tagged vector control or redox mutant C65S/C99S APE1 expressing plasmid constructs. Twenty-four hours after transfection, cells were immunostained with α-1H6 antibody and visualized by confocal microscopy (magnification 63×; scale bars: 10 μm). **(B)** APE1 levels in these cell extracts were examined by western blot analysis with α-APE1 and α-HSC70 (loading control) antibodies. **(C)** PLA was performed in APE1 KD MIA PaCa-2^APE1shRNA^ cells expressing control vector or C65S/C99S APE1 mutant with antibodies against APE1, G4 and PLA probes. G4–APE1 PLA foci were visualized by confocal microscopy images (magnification: 63×; scale bars: 10 μm). **(D)** Quantitation of the number of PLA puncta per cell (*n* = 50 cells). **(E)** MIA PaCa-2 cells were treated with increasing doses of APE1 redox inhibitor E3330 for 6 h and then immunostained with α-1H6 and α-APE1 antibodies and visualized by confocal microscopy. Cells were counterstained with DAPI (magnification: 63×; scale bars: 10 μm). **(F)** Relative *KRAS* gene expression in MIA PaCa-2^APE1shRNA^ cells expressing C65S/C99S APE1 plasmid by qRT-PCR assays. Fold change (normalized to GAPDH) was calculated. The *P-*values were determined using unpaired Student’s *t*-tests (****P< 0.0001, **P< 0.01, n.s. (nonsignificant) = *P*> 0.05). Error bars denote ±SD. Three independent experiments were performed.

So far, our data suggest that APE1 binds to *KRAS* G4 structure and promotes the binding of MAZ and PARP1 to the promoter region to activate gene *KRAS* expression. Since APE1 has been shown to stimulate the DNA-binding activity of a number of TFs through its redox function, thereby regulating gene expression (38,44), we investigated whether the redox function of APE1 is involved in regulation of G4 formation and G4-mediated *KRAS* expression. Using immunofluorescence studies, we observed that the loss of G4 staining in APE1 KD MIA PaCa-2 cells can be rescued by re-introduction of the APE1 redox mutant C65S/C99S (70,71) in these cells, indicating that the redox function of APE1 is not involved in the regulation of the G4 structure formation (Figure 5A and B). Furthermore, our G4:APE1 PLA experiment with the C65S/C99S APE1 revealed appreciable number of PLA puncta indicating that the APE1 redox mutant has the ability to interact with G4 structures in the cells (Figure 5C and D). To further confirm that the redox function of APE1 is not involved in G4 regulation, we used APE1 redox inhibitor, E3330, and performed G4 and APE1 immunofluorescence studies. Small molecule E3330 has been extensively characterized as a direct and selective inhibitor of the redox function of APE1 (72–74). We observed no change in the G4 staining upon treating the cells with increasing doses of E3330, thus confirming that the redox function of APE1 is not involved in the regulation of G4 formation in cells (Figure 5E). We also examined the effect of redox mutant C65S/C99S APE1 on the *KRAS* expression using the qRT-PCR assay. Our analysis indicated no significant change in the *KRAS* expression in C65S/C99S APE1 transfected cells when compared to the control cells, indicating that the redox function is not involved in regulating *KRAS* expression (Figure 5F).

### Downregulation of APE1 sensitizes PDAC cells to chemotherapy *in vitro* and suppresses xenograft tumor growth *in vivo*

**Figure 6.**
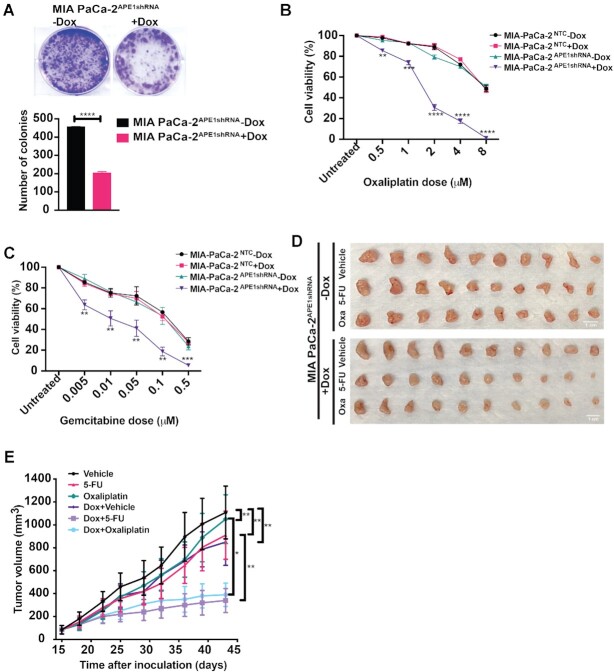
APE1 KD sensitizes PDAC cells to chemotherapy *in vitro* and suppresses xenograft tumor growth *in vivo*.**(A)** MIA PaCa-2^APE1shRNA^ cells were treated with or without Dox and the effect of APE1 KD on cell survival was evaluated using a colony formation assay. An unpaired Student’s *t*-test comparing the number of colonies in control versus APE1 KD cells was used to determine the *P-*value (****P< 0.0001). MIA PaCa-2^APE1shRNA^ and MIA PaCa-2^NTC^ cells treated without or with Dox for 4 days and then exposed to indicated doses of oxaliplatin **(B)** or gemcitabine **(C)**. The graphs show the percentage cell viability obtained from colony formation assays, performed three times in triplicates. **(D)** MIA PaCa-2^APE1shRNA^ cells were subcutaneously implanted in nude mice. When palpable tumors were visible, mice were divided into six groups (*n* = 5 in each group). Three groups were fed with Dox (2 mg/ml) and other three groups without Dox containing drinking water following which 5-FU (30 mg/kg) or oxaliplatin (2 mg/kg) were injected intraperitonially three times a week for 4 weeks and tumor volume was recorded using a caliper. Resected tumors after completion of treatment are shown. **(E)** Tumor volume was measured at indicated days and tumor growth curve was plotted. The *P-*values were determined using an unpaired Student’s *t*-test (****P< 0.0001, ***P< 0.001, **P< 0.01, *P< 0.05). Error bars denote ±SD.

Being a multifunctional protein with DNA damage repair, redox function and transcriptional regulatory roles, APE1 has been shown to promote cell survival and resistance to many chemotherapeutic drugs (75–77). *In vitro* colony formation assays revealed that APE1 KD in MIA PaCa-2 cells significantly reduced the number of colonies compared to the control MIA PaCa-2 cells (Figure 6A). Next, we challenged the APE1 KD cells with increasing doses of chemotherapeutic drugs 5-FU, oxaliplatin or gemcitabine that are routinely used for the treatment of PDAC. Several studies have shown that these drugs can exert their cytotoxicity by multiple mechanisms, including generation of oxidative base damage lesions and abasic sites in the DNA that are repaired by the APE1-mediated BER pathway (78–80). Further, expression of WT or mutant *KRAS* was also found to be associated with promoting resistance to these drugs in colon cancer cells and patients (81,82). We found KD of APE1 resulted in significant increases in sensitivity of MIA PaCa-2 cells to oxaliplatin (Figure 6B), gemcitabine (Figure 6C) and 5-FU (Supplementary Figure S5A). Similar increases in sensitivity to these chemotherapeutic agents were also observed upon APE1 KD in PANC-1 (Supplementary Figure S5B and C) and BxPC-3 cells (Supplementary Figure S5D–F) when compared to the control cells. These data together suggest that targeting APE1 represents an attractive approach to sensitize PDAC cells to chemotherapy.

To examine whether KD of APE1 can also enhance sensitivity to 5-FU and oxaliplatin and suppress PDAC tumor growth *in vivo*, we utilized a tumor xenograft model with MIA PaCa-2^APE1shRNA^ cells. When palpable tumors were visible, mice were fed drinking water without Dox or with Dox (2 mg/ml) to downregulate APE1 levels. Then, the effect of 5-FU and oxaliplatin on tumor growth of MIA PaCa-2^APE1shRNA^ cells was evaluated. While treatment with either drug alone showed moderate effect on the tumor growth in control (without Dox) mice as indicated by the tumor volume, the same treatment significantly reduced the tumor growth of MIA PaCa-2^APE1shRNA^ cells in mice fed with Dox, demonstrating that KD of APE1 sensitizes PDAC cell growth *in vivo* (Figure 6D and E). Further, IHC analysis confirmed the KD of APE1 level in the tumor tissues of mice fed with Dox (Supplementary Figure S5G). Collectively, our data demonstrate that KD of APE1 sensitizes PDAC cells to routinely used chemotherapy drugs both *in vitro* and *in vivo*.

### Elevated levels of APE1 and G4 DNA are present in PDAC patient’s tissue samples

**Figure 7.**
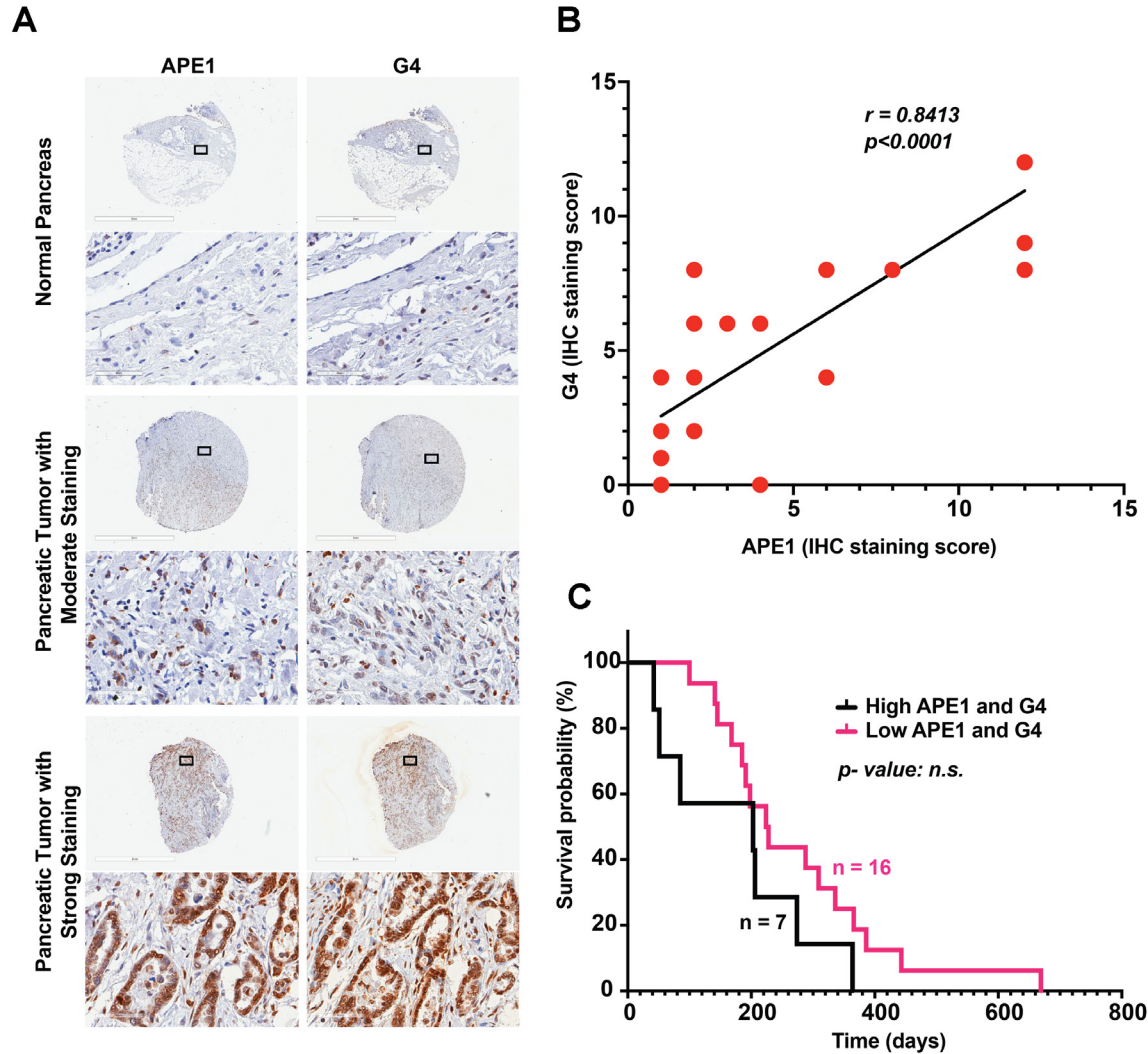
Elevated levels of APE1 and G4 DNA in PDAC patients’ tissue samples. **(A)** IHC analysis of APE1 and G4 levels in normal and PDAC patients’ tissue samples (magnification: 2× and 40×; scale bars: 2 mm and 60 μm). The percentage of positive staining and the intensity were examined as described in the ‘Materials and Methods’ section. **(B)** The correlation between APE1 and G4 staining in the tissue samples was analyzed using simple linear regression. **(C)** The overall survival of PDAC patients in relation to APE1 and G4 staining was analyzed by Kaplan–Meier analysis.

Given that APE1 is often overexpressed in multiple types of cancers including PDAC (47), and our current observation that APE1 regulates G4 DNA formation and promotes *KRAS* expression, we determined the levels of APE1 and G4 DNA in 23 PDAC patients’ tissue samples and 2 normal control pancreas tissues using IHC analysis with APE1 and G4 structure-specific antibodies. PDAC patient TMAs were obtained from the Rapid Autopsy Program for Pancreas at UNMC. Among these samples, 18 patients were at stage IV, 1 at stage III and 4 at stage II. IHC scoring based on percentage of positively staining cells and staining intensity revealed that APE1 and G4 DNA levels were significantly higher in PDAC tumor tissues compared to normal controls (Figure 7A). Further analysis revealed a strong positive correlation between APE1 and G4 staining in these tissue samples (Spearman’s *r* = 0.8413, *P* < 0.0001), specifically in stage IV tumors (Figure 7B). This indicates that increased APE1 levels are associated with elevated levels of G4 DNA in highly aggressive PDAC tumors.

To determine the clinical significance of elevated levels of G4 and APE1 in PDAC, we extended our analyses by correlating APE1 and G4 DNA levels with the survival of the patients who were administered chemotherapies including gemcitabine, capecitabine, leucovorin-modulated 5-FU, oxaliplatin, etc. Our survival analysis showed that among the 23 PDAC patients, 7 patients with the highest IHC staining score for APE1 and G4 survived for fewer days compared to the rest with lower staining scores (Figure 7C). However, due to the limitation in the number of patient’s tissue samples, we were unable to find a statistically significant difference between the two groups. Nonetheless, these data provide preliminary evidence that the expression levels of APE1 and G4 DNA negatively correlate with PDAC patients’ prognosis.

## DISCUSSION

Over the past decade, G4 structures have gained recognition as genomic features involved in *KRAS* transcriptional regulation (30,83). However, little is known about the factors that regulate the formation of stable G4 structures in the *KRAS* promoter in PDAC cells. In this study, we have demonstrated that APE1 plays a crucial role in the formation of stable G4 structures and regulating *KRAS* expression in PDAC cells. Loss of APE1 abrogated the formation of G4 structures and the loading of TFs such as MAZ and PARP1 on the *KRAS* promoter, resulting in reduced *KRAS* expression in multiple PDAC cell lines. Further, by demonstrating that APE1 can strongly bind to *KRAS* promoter G4 structure and stabilize the folding *in vitro*, we identified APE1 as a G4-binding protein. Supporting this idea, we have provided evidence that APE1 colocalizes and interacts with G4 structures in cells. Importantly, using PDAC patients’ tissue samples we have demonstrated higher APE1 and G4 DNA levels in the tumor tissues compared to control pancreatic tissues. Therefore, our study has identified and characterized APE1 as a key regulator of the formation of stable G4 structures in the genome and advanced our understanding of the mechanism underlying G4 regulation and G4-mediated transcriptional control of *KRAS* in PDAC cells. Given that APE1 is overexpressed in PDAC (47) and G4s are known to regulate cancer driver genes (23), our findings also implicate that G4 can be considered as a novel prognostic marker and promising therapeutic target in PDAC.

APE1 is a multifunctional protein with DNA damage repair and transcriptional regulatory Ref-1 functions (84). Its primary function in the repair of spontaneously generated AP sites in the genome via the BER pathway and its role in regulating gene expression via redox function (APE1 reduces the oxidized cysteine in many TFs) have been well established (37,38). In this study, we have discovered a novel and noncanonical function of APE1 in regulating gene expression through regulating the formation of DNA secondary structures, G4s. Recent ChIP sequencing using a G4 structure-specific antibody has revealed that endogenous G4s are enriched in nucleosome-depleted promoter/enhancer regions upstream of TSSs (21). Furthermore, most of the endogenous G4s are hubs for binding of TFs (22). Thus, it is possible that elevated gene expression results from increased TF loading onto the promoter G4s and recruitment of RNA Pol II. A recent study has demonstrated that promoter G4 folding leads to the retention of RNA Pol II, suggesting that G4s act as a site for the recruitment of key components of the transcriptional machinery as well as TFs (85). This is supported by the observation that several TFs, including SP1 (86), CNBP (87), PARP1 (88), MAZ (89) and LARK (90), display high-affinity binding for G4s *in vitro*. Consistent with this, multiple previous studies from Xodo’s group have established that binding of MAZ and PARP1 to *KRAS* promoter G4 plays a key role in activation of *KRAS* expression (69,89). Here, we add to the current knowledge by providing compelling evidence that APE1 regulates *KRAS* expression through a mechanism that involves facilitating the formation of promoter G4 structure and loading of the TFs such as MAZ and PARP1 to the promoter region. Several lines of evidence were provided to support this: (i) APE1 colocalizes and interacts with G4 structures in cells, and recombinant APE1 can directly bind *KRAS* promoter G4 structure *in vitro*; (ii) downregulation of APE1 reduces stable G4 formation, loading of MAZ and PARP1 on *KRAS* promoter, and *KRAS* expression; and (iii) G4-stabilizing ligands fail to activate *KRAS* expression in APE1 downregulated cells, suggesting APE1 is required for G4-mediated *KRAS* expression.

**Figure 8.**
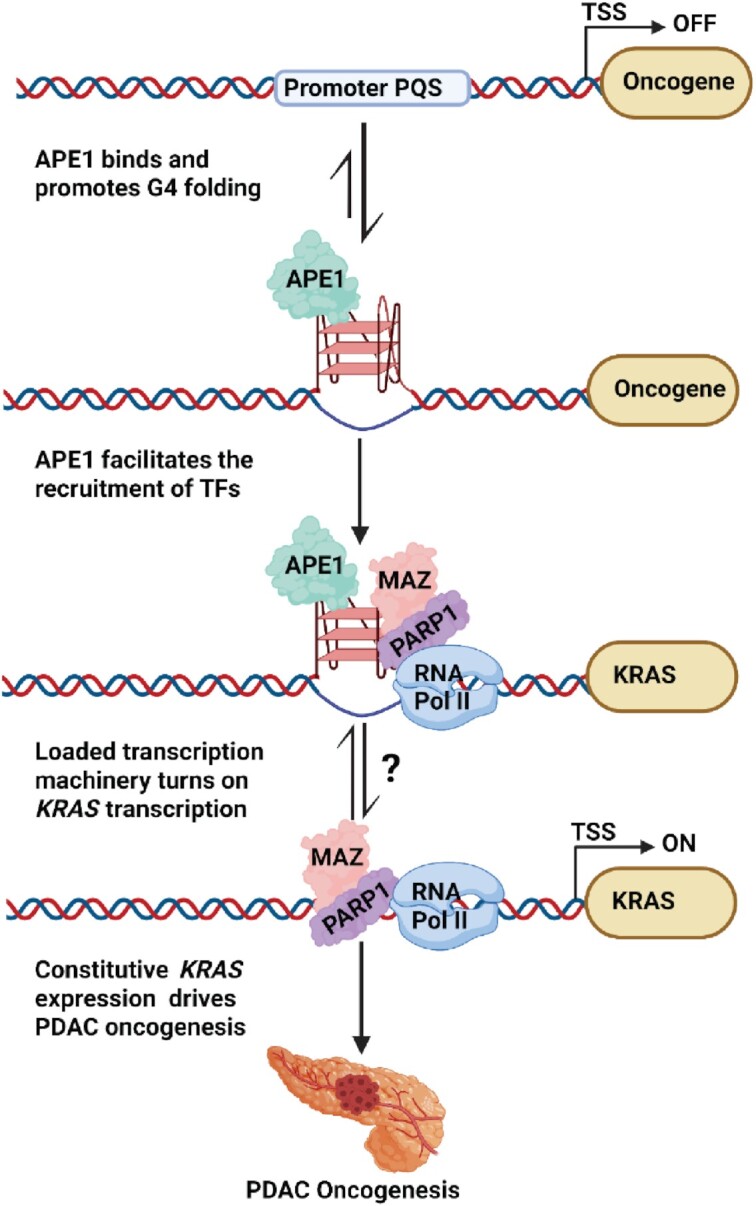
A model for APE1-mediated regulation of G4 formation and loading of the TFs to drive *KRAS* expression (created with BioRender.com).

The exact mechanism by which APE1 regulates the formation of stable G4 in cells is not fully understood. Active transcription-induced torsional stress and negative superhelicity had been proposed to stimulate G4 formation at promoters (91,92). However, a recent study has demonstrated that folding of G4s in promoters does not necessarily require active transcription but can be favored by an accessible open chromatin state (85). This raises the possibility that potential quadruplex sequences (PQSs) in open chromatin promoter regions are in dynamic equilibrium between double-stranded and transient G4 folding forms. We propose that binding of APE1 to transiently formed G4 may shift the equilibrium toward a stable G4 structure. Consistent with this, our recent genome-wide binding analysis revealed that APE1 is highly enriched in promoter enhancer regions that overlap with G4s (35). Although low-resolution mapping (300 bp) demonstrated significant overlaps between AP sites and G4s (35), our current study demonstrates that recombinant APE1 has high affinity for binding to a G4 structure without any AP site, suggesting that an AP site is not a prerequisite for APE1 to bind to G4. Therefore, we postulate that the PQS in open chromatin region transiently generates G4 and binding of APE1 promotes the formation of a stable G4 structure. The stable G4 structure acts as a loading platform for the TFs MAZ and PARP1 that subsequently drive *KRAS* expression (Figure 8). Binding of APE1 to noncanonical (non-B-form) DNA secondary structures is not unprecedented. APE1 was shown to recognize many non-B-form DNA structures such as cruciform DNA that is formed by a palindromic core sequence (TGAGACAGGGTCTCA) known as negative calcium response element sequence (nCaRE), present in many gene promoters (93,94). Indeed, previous studies from our lab and others have shown APE1-dependent regulation of nCaRE-B containing human PTH, renin and SIRT1 gene promoters (43,93–95). Thus, it appears that APE1 binds and promotes the formation of stable G4 structures in promoter/enhancer regions of many genes that provide a loading platform for the recruitment of TFs to activate gene expression. Further studies examining how CRISPR/Cas9-mediated deletions or mutations of G4-forming sequences on the promoter of *KRAS* gene affect the recruitment of TFs and *KRAS* expression are warranted.

Many earlier studies have shown that treatment of cells with G4-stabilizing ligands reduces *KRAS* expression and thus portrayed G4s as antagonists to *KRAS* gene expression (17,30). In contrast, our results have demonstrated that treatment with G4-stabilizing small molecules PDS and TMPyP4 for a shorter duration (2 h) activates *KRAS* expression in PDAC cells, suggesting G4 as a positive regulator for *KRAS* expression. The apparent discrepancy between these results can be explained by the fact that the previously published studies have relied on the effect of prolonged treatment with these ligands (12 h or more), which induced DNA double-strand breaks that negatively affect transcription (96). It was shown that treatment with the potent G4 ligand PDS caused DNA damage at G4 sites in a transcription- and replication-dependent fashion (97). Similarly, Hurley and coworkers reported that prolonged treatment with G4 ligands could lead to *MYC* downregulation by an indirect consequence of the ligands eliciting global G4 stabilization (98). Our study showed that APE1-mediated formation of stable G4 structure activated *KRAS* expression. In support of this, emerging evidence suggests that G4s can act as positive regulators of transcription by multiple mechanisms (99). This includes the role of G4s in mediating the placement of histone marks and in interacting with chromatin remodeling proteins, thus facilitating TF and RNA Pol II loading (85). Additionally, G4s have a positive effect on transcription by stabilizing R-loop formation (100). G4s can influence long-distance gene regulation by promoting distal interactions between enhancers and promoters (101).

APE1 is overexpressed in PDAC, and elevated levels of APE1 and its redox function in PDAC have been shown to promote tumor cell survival, migration and angiogenesis, and confer resistance to chemotherapeutic drugs (47,56,102). We have shown that downregulation of APE1 inhibited tumor growth and sensitized these cells to conventional chemotherapy drugs gemcitabine, 5-FU and oxaliplatin both *in vitro* and in a *in vivo* xenograft model. Even though oxaliplatin exerts its cytotoxicity primarily by inducing DNA intra- or interstrand cross-links that are repaired by the nucleotide excision repair pathway (103), studies have shown that treatment with platinum-based drugs cisplatin and oxaliplatin produces reactive oxygen species that damage DNA by oxidizing DNA bases in cells (78,79). APE1 plays a key role in the repair of these damaged bases via the BER pathway. Similarly, 5-FU or its metabolites that primarily inhibit thymidylate synthase (104), the key enzyme of *de novo* pyrimidine biosynthesis, can also directly incorporate into genomic DNA. A previous study has shown that uracil DNA glycosylase SMUG1 efficiently removes 5-FU from DNA *in vitro* and in cells to generate AP sites that are repaired by APE1-mediated BER (80,105). Furthermore, APE1 redox function was also shown to confer 5-FU resistance in colon cancer because treatment with APE1 redox inhibitor E3330 enhanced tumor response to 5-FU in a colon cancer xenograft model (54). Our previous studies and others showed that APE1 downregulation sensitizes colon and breast cancer cells to 5-FU and cisplatin/oxaliplatin *in vitro* (106,107). Since the expression of WT or mutant *KRAS* has also been shown to be associated with resistance to these drugs in cancer (81,82), our study implicates that APE1-mediated *KRAS* expression may contribute to promoting resistance to these drugs in PDAC. Therefore, we propose that elevated levels of APE1 promote proliferation and drug resistance in PDAC by facilitating repair of damaged bases, activating gene expression through its redox function and controlling G4-mediated *KRAS* expression.

Using rapid autopsy tissue samples from PDAC patients, we demonstrated for the first time a correlation between APE1 levels and G4 DNA in human PDAC tissue specimens. This observation has important implication for regulatory functions of APE1 in tumor progression, as cancer cells have increased levels of G4 DNA and APE1, both of which regulate expression of cancer driver genes. Elevated G4 staining in tumor tissues of stomach and liver cancer patients suggested that cancer cells have increased levels of G4 DNA (108). Notably, recent genome-wide mapping of G4 DNA forming regions in 22 breast cancer patient-derived tumor xenografts revealed that G4s are significantly enriched in promoters of highly expressed genes when compared to those with medium or low expression (109). Furthermore, increased G4 DNA is associated more often with the signature cancer-driving genes in aggressive triple negative breast cancer, as compared to other subtypes. We propose that APE1 plays a crucial role in PDAC oncogenesis by binding and promoting the formation of stable G4 structures in promoter/enhancer regions, which in turn facilitate the loading of TFs onto the promoters to regulate G4-mediated gene expression. Thus, identification of small molecules that bind to APE1 and affect its interaction with G4 could be a promising strategy for PDAC therapy.

In conclusion, our study has identified APE1 as a G4 DNA-binding protein and key regulator of stable G4 formation, and unraveled G4–APE1 functional complex as a major determinant for *KRAS* oncogene regulation in PDAC, underscoring a novel therapeutic opportunity.

## Supplementary Material

gkac172_Supplemental_File
