# Supplementary material for: The human AP-endonuclease 1 (APE1) is a DNA G-quadruplex structure binding protein and regulates KRAS expression in pancreatic ductal adenocarcinoma cells
Source: Nucleic Acids Res. 2022 Mar 14;50(6):3394–412. doi: 10.1093/nar/gkac172 (PMC8990529; doi:10.1093/nar/gkac172)
Supplement: gkac172_Supplemental_File [file gkac172_supplemental_file.pdf]

Supplementary Data

The Human AP-endonuclease 1 (APE1) is a DNA G-quadruplex Structure Binding Protein and Regulates *KRAS* Expression in Pancreatic Ductal Adenocarcinoma Cells

Suravi Pramanik[1], Yingling Chen[1], Heyu Song[1], Irine Khutsishvili[2], Luis A. Marky[2], Sutapa Ray[3], Amarnath Natarajan [4,5], Pankaj K. Singh[4,5], and Kishor K. Bhakat [1,5]*

[1] Department of Genetics, Cell Biology and Anatomy, University of Nebraska Medical Center, Omaha, NE, USA 68198

[2] Department of Pharmaceutical Sciences, University of Nebraska Medical Center, Omaha, NE, USA 68198

[3] Department of Pediatrics, Hematology/Oncology division, University of Nebraska Medical Center, Omaha, NE, USA 68198

[4] Eppley Institute for Research in Cancer and Allied Health, University of Nebraska Medical Center, Omaha, NE, USA 68198

[5] Fred & Pamela Buffett Cancer Center, University of Nebraska Medical Center, Omaha, NE, USA 6819

Contents:

Figure S1: APE1 plays a crucial role in the formation of G4 structures in PDAC cells.

Figure S2: APE1 interacts with G4 structures in cells and APE1 knockdown (KD) specifically abrogates G4 staining.

Figure S3: APE1 N-terminus 1-42 amino acid peptide cannot bind to *KRAS* promoter G4 structure.

Figure S4: G4 ligands PDS and TMPyP4 bind to *KRAS* promoter G4 with high affinity and APE1 regulates *KRAS* expression in PDAC cells.

Figure S5: APE1 KD sensitizes PDAC cells to chemotherapy.

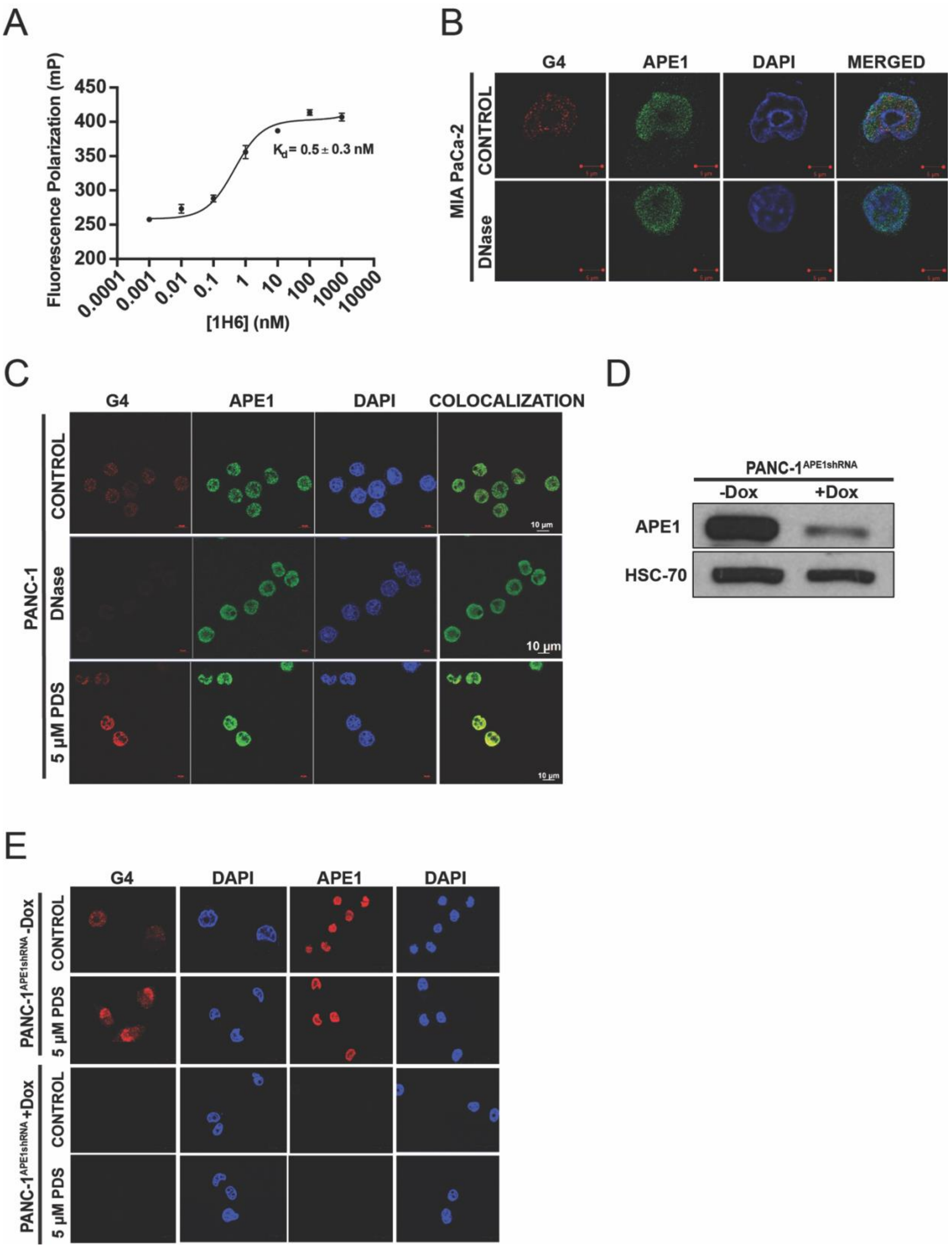
A
Fluorescence Polarization (mP)
450
400
350
300
250
200
0.0001
0.001
0.01
0.1
1
10
100
1000
10000
[1H6] (nM)
Kd = 0.5 ± 0.3 nM
B
G4
APE1
DAPI
MERGED
MIA PaCa-2
CONTROL
DNase
C
G4
APE1
DAPI
COLOCALIZATION
PANC-1
CONTROL
DNase
5 µM PDS
10 µm
D
PANC-1APE1shRNA
-Dox
+Dox
APE1
HSC-70
E
G4
DAPI
APE1
DAPI
PANC-1APE1shRNA -Dox
PANC-1APE1shRNA +Dox
CONTROL
5 µM PDS

Figure S1. APE1 plays a crucial role in the formation of G4 structures in PDAC cells. **(A)** Changes in Fluorescence Polarization (FP) of 6-FAM-labeled G4-forming KRAS oligo (10 nM) incubated with G4-structure specific antibody, clone 1H6, over the range of concentrations is shown. The dissociation constant (K_d) was calculated (nonlinear regression (curve-fit)-total saturation binding on Prism 8.0.) from three independent experiments with triplicates samples and plotted using Prism 8.0; average ±SD are shown in the graph. **(B)** SIM images of MIA PaCa-2 cells treated without or with DNase, immunostained with α-1H6 and α-APE1 antibodies and counterstained with DAPI (Magnification: 63X, Scale bars: 5 µm). **(C)** Confocal microscopy images of PANC-1 cells treated with DNase or PDS, immunostained with α-1H6 and α-APE1 antibodies and counterstained with DAPI (Magnification: 63X, Scale bars: 10 µm). **(D)** PANC-1 cells expressing APE1shRNA under Dox-inducible promoter were treated without or with 2 µg/mL Dox for 4 days and APE1 levels were examined by Western blot using α-APE1 and α-HSC70 (loading control) antibodies. **(E)** PANC-1^{APE1shRNA} cells treated without or with Dox and without or with PDS were immunostained with α-1H6 and α-APE1 antibodies and visualized by confocal microscopy (Magnification: 63X, Scale bars: 20 µm). Three independent experiments were performed for each immunofluorescence-based study.

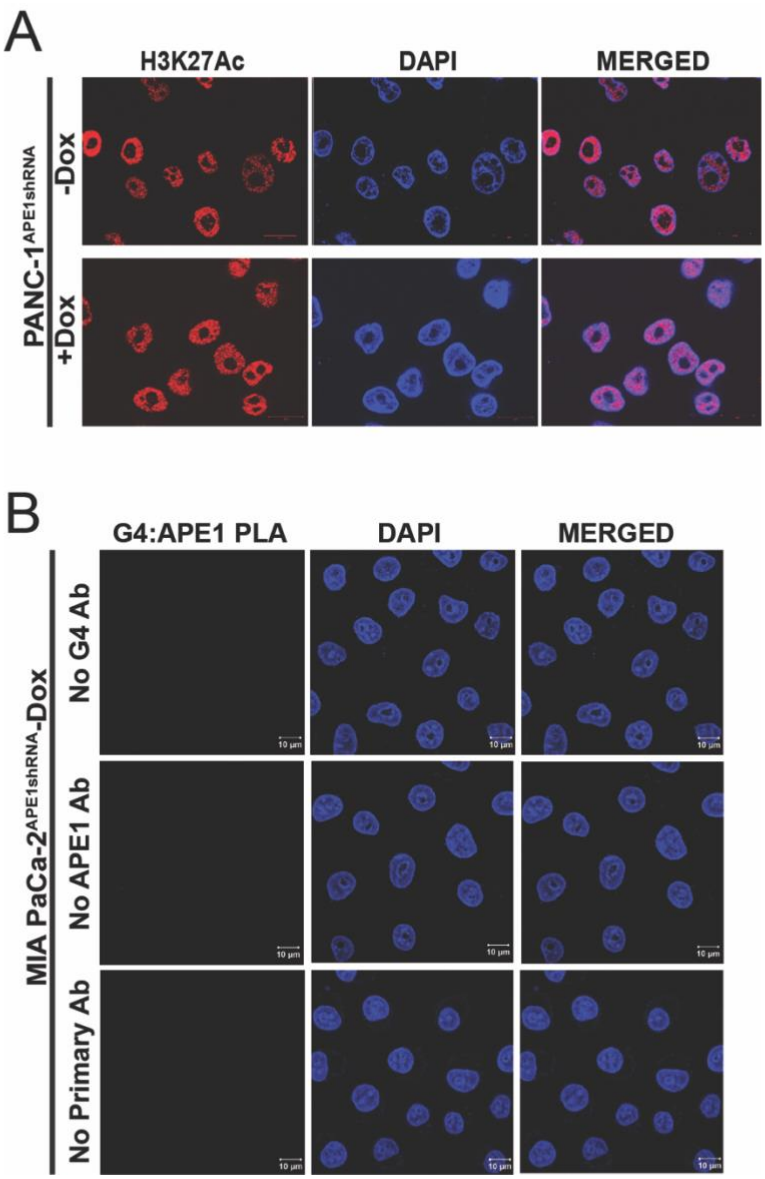

Figure S2. APE1 interacts with G4 structures in cells and APE1 knockdown (KD) specifically abrogates G4 staining. **(A)** PANC-1 ^{APE1shRNA} cells were treated without (top) or with (bottom) Dox and immunostained with α-H3K27Ac antibody and visualized by confocal microscopy (Magnification: 63X, Scale bars: 20 µm). **(B)** Confocal microscopy images of MIA PaCa-2^{APE1shRNA} cells immunostained with primary antibody either against APE1 or against G4 or no primary antibody and secondary PLA probes. G4:APE1 PLA foci were visualized by confocal microscopy (Magnification: 63X, Scale bars: 10 µm).

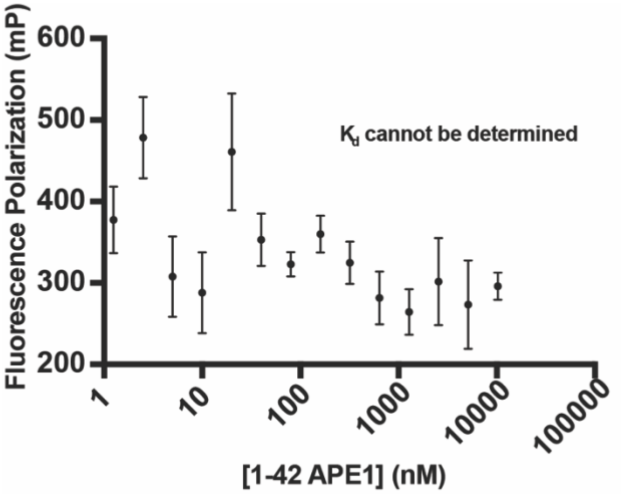

Figure S3. APE1 N-terminus 1-42 amino acid peptide cannot bind to *KRAS* promoter G4 structure. 6-FAM-labeled G4-forming KRAS oligo (10 nM) was incubated with increasing concentrations of recombinant GST-tagged APE1 N-terminus 1-42 amino acid peptide in a buffer containing 50 mM KCl, 50 mM Tris-HCl pH 7.5, 1 mM $MgCl_2$, 1 mM DTT, and 0.1 mM EDTA and Fluorescence Polarization (FP) values were recorded. The data points from three independent experiments with triplicate samples were plotted using Prism 8.0.

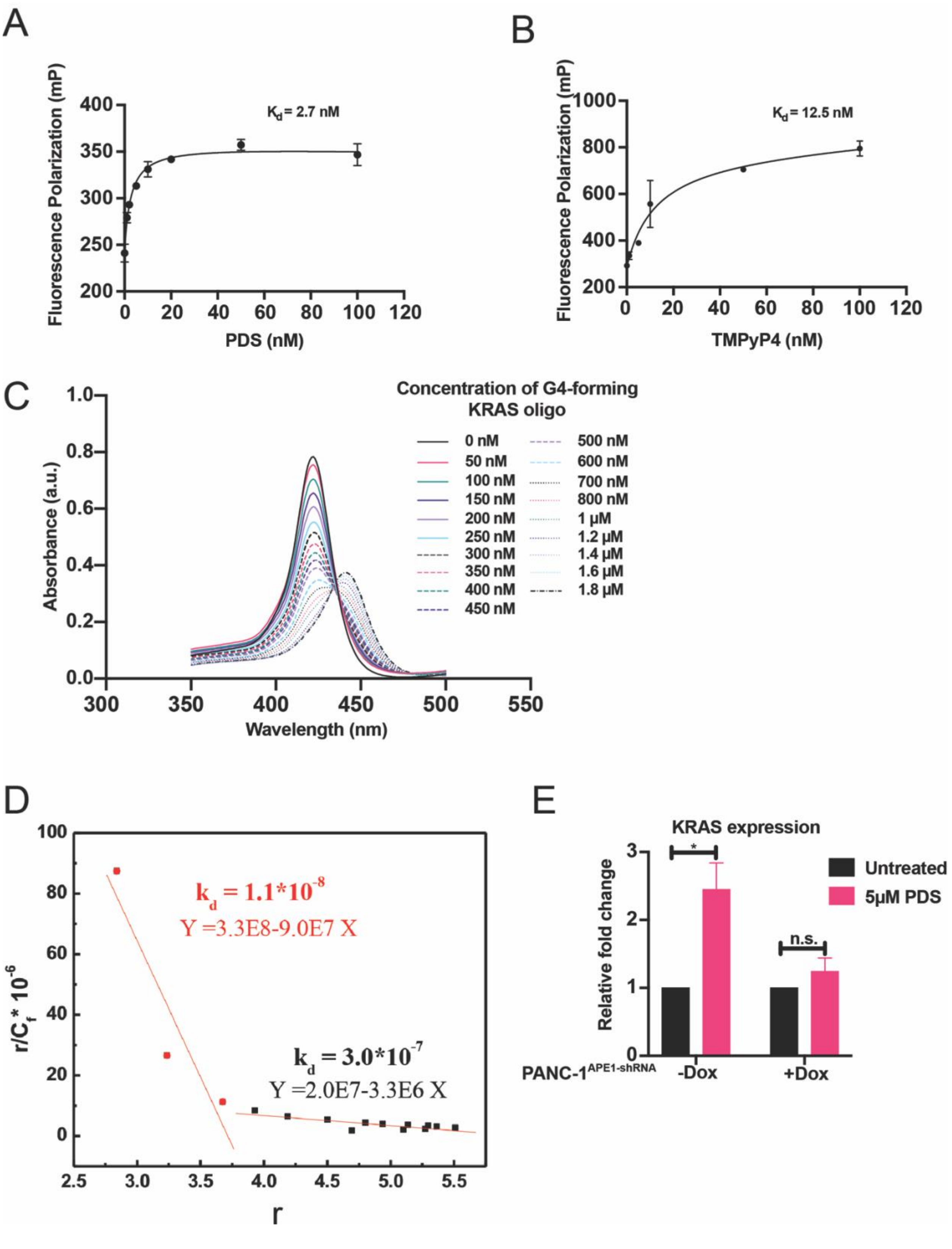

Figure S4. G4 ligands PDS and TMPyP4 bind to *KRAS* promoter G4 with high affinity and APE1 regulates *KRAS* expression in PDAC cells. **(A-B)** FP assays were performed

with 6-FAM-labeled G4-forming KRAS oligo (10 nM) and increasing concentrations of PDS **(A)** or TMPyP4 **(B)**. FP values were measured from 3 independent experiments performed with triplicate samples and plotted as a function of ligand concentration using Prism 8.0. The dissociation constant (K_d) was calculated (nonlinear regression (curve-fit)-total saturation binding on Prism 8.0). **(C)** UV-Vis absorption titration spectra of TMPyP4 (4 µM) solution incubated in a buffer containing 10 mM Tris-HCl pH 7.5, 1 mM EDTA and 50 mM KCl. alone or in presence of increasing concentrations of G4-forming KRAS oligo (0 nM- 1.8 µM). **(D)** Scatchard plot showing the dissociation constant (K_d) for specific (red) and non-specific (black) binding of TMPyP4 to the G4-forming KRAS oligo. Consistent results were obtained in three independent experiments. **(E)** qRT-PCR assay was performed using PANC-1^{APE1shRNA} cells treated without and with Dox and with or without 5 µM PDS. Relative *KRAS* gene expression (normalized to GAPDH) was calculated. The *p*-values were determined using unpaired Student's t test (**p*<0.05, n.s. (nonsignificant)= *p*>0.05). Error bars denote ±SD. Experiments were performed thrice in triplicates.

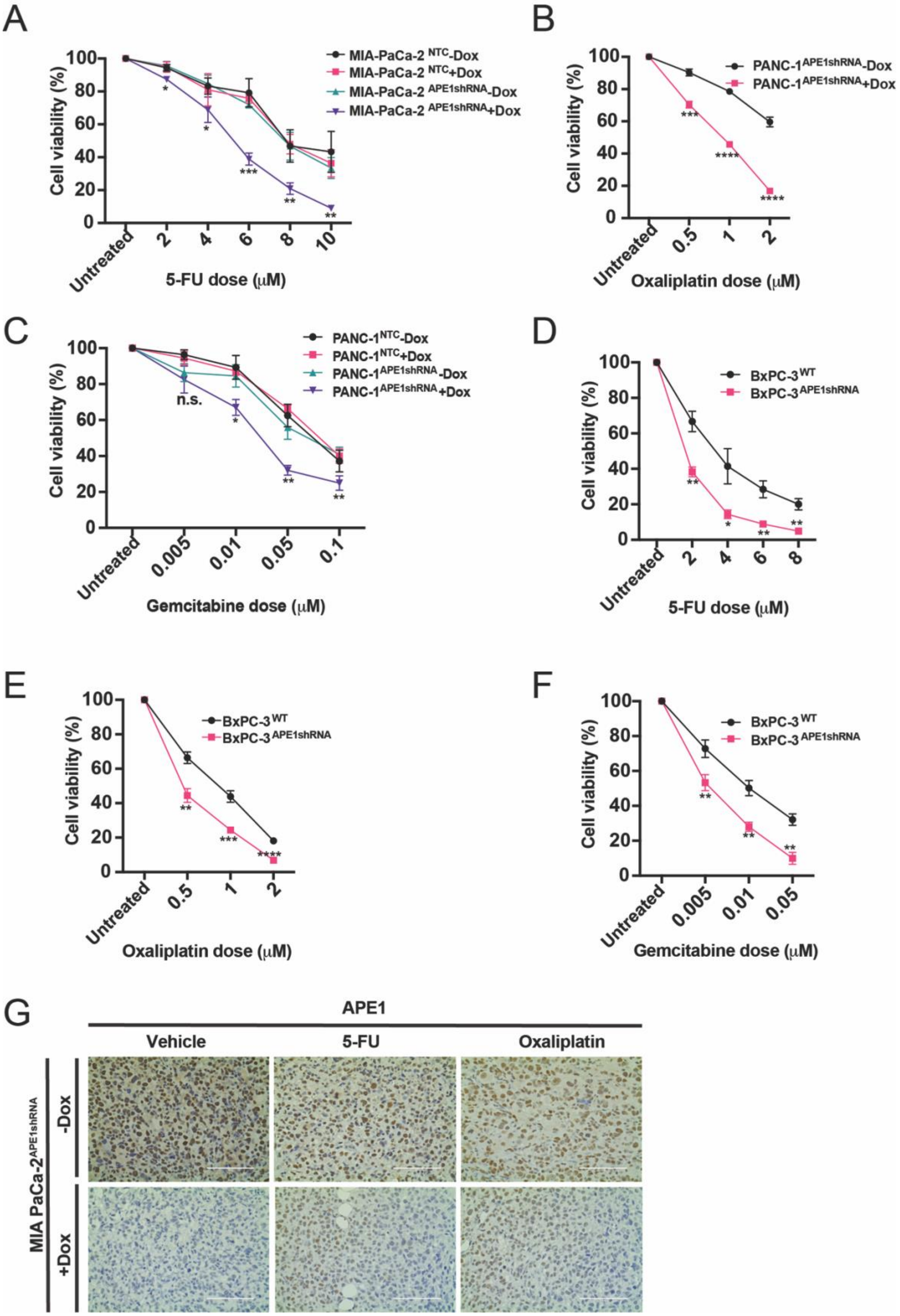
A
Cell viability (%)
5-FU dose (μM)
Untreated
MIA-PaCa-2 NTC-Dox
MIA-PaCa-2 NTC+Dox
MIA-PaCa-2 APE1shRNA-Dox
MIA-PaCa-2 APE1shRNA+Dox
B
Oxaliplatin dose (μM)
PANC-1APE1shRNA-Dox
PANC-1APE1shRNA+Dox
C
Gemcitabine dose (μM)
PANC-1NTC-Dox
PANC-1NTC+Dox
PANC-1APE1shRNA-Dox
PANC-1APE1shRNA+Dox
n.s.
D
5-FU dose (μM)
BxPC-3WT
BxPC-3APE1shRNA
E
Oxaliplatin dose (μM)
BxPC-3WT
BxPC-3APE1shRNA
F
Gemcitabine dose (μM)
BxPC-3WT
BxPC-3APE1shRNA
G
APE1
Vehicle
5-FU
Oxaliplatin
MIA PaCa-2APE1shRNA
-Dox
+Dox

Figure S5. APE1 KD sensitizes PDAC cells to chemotherapy. **(A)** MIA PaCa-2^{APE1shRNA} and MIA PaCa-2^{NTC} cells treated without or with Dox for 4 days and then exposed to indicated doses of 5-FU. The graph shows the percentage cell viability obtained from the colony formation assays. **(B-C)** Control and APE1 KD PANC-1 cells treated with indicated doses of oxaliplatin **(B)** and gemcitabine **(C).** The graphs show the percentage cell viability obtained from the colony formation assays. **(D-F)** Colony formation assays performed using BxPC-3^{WT} and BxPC-3^{APE1shRNA} cells treated with indicated doses of 5-FU **(D)** or oxaliplatin **(E)** or gemcitabine **(F).** All colony formation assays were performed thrice in triplicates. The *p*-values were determined using unpaired Student's t test (**** $p<0.0001$, ***$p<0.001$, **$p<0.01$, *$p<0.05$, n.s. (nonsignificant)= $p>0.05$). Error bars denote ±SD.**(G)** Tumor xenograft sections from control and treatment groups were immunostained with anti-APE1 antibody (Magnification: 20X, Scale bars: 100 µm).
